# Supplementary material for: Health professionals’ experiences and views on obstetric ultrasound in Vietnam: a regional, cross-sectional study
Source: BMJ Open. 2019 Sep 22;9(9):e031761. doi: 10.1136/bmjopen-2019-031761 (PMC6773349; doi:10.1136/bmjopen-2019-031761)
Supplement: Author's manuscript [file bmjopen-2019-031761.draft_revisions.pdf]

BMJ Open is committed to open peer review. As part of this commitment we make the peer review history of every article we publish publicly available.

When an article is published we post the peer reviewers' comments and the authors' responses online. We also post the versions of the paper that were used during peer review. These are the versions that the peer review comments apply to.

The versions of the paper that follow are the versions that were submitted during the peer review process. They are not the versions of record or the final published versions. They should not be cited or distributed as the published version of this manuscript.

BMJ Open is an open access journal and the full, final, typeset and author-corrected version of record of the manuscript is available on our site with no access controls, subscription charges or pay-per-view fees (http://bmjopen.bmj.com).

If you have any questions on BMJ Open's open peer review process please email info.bmjopen@bmj.com

BMJ Open

Health professionals' experiences and views on obstetric ultrasound in Vietnam: a regional, cross-sectional study

Journal:	*BMJ Open*
Manuscript ID	bmjopen-2019-031761
Article Type:	Original research
Date Submitted by the Author:	20-May-2019
Complete List of Authors:	Holmlund, Sophia; Umea Universitet Medicinska fakulteten, Clinical Sciences, Obstetrics and Gynecology<br>Pham Thi, Lan; Hanoi Medical University, Department of Dermatology and Venereology<br>Edvardsson, Kristina; Umea Universitet Medicinska fakulteten, Department of Clinical Sciences, Obstetrics and Gynecology; La Trobe University, Judith Lumley Centre, School of Nursing and Midwifery<br>Ho Dang, Phuc ; Vietnam Academy of Science and Technology, Department of Probability and Mathematical Statistics, Institute of Mathematics<br>Ntaganira, Joseph; University of Rwanda College of Medicine and Health Sciences, School of Public Health<br>Small, Rhonda; La Trobe University, Judith Lumley Centre, School of Nursing and Midwifery; Karolinska Institutet, Department of Women's and Children's and Reproductive Health<br>Lesio Kidanto, Hussein; Aga Khan University - Tanzania, Department of Obstetrics and Gynecology<br>Ngarina, Matilda; Muhimbili National Hospital, Department of Obstetrics and Gynecology<br>Mogren, Ingrid; Umeå Universitet Medicinska fakulteten, Department of Clinical Sciences, Obstetrics and Gynecology; La Trobe University, Judith Lumley Centre, School of Nursing and Midwifery
Keywords:	prenatal ultrasonography, obstetricians, midwives, prenatal care, pregnancy, Vietnam

SCHOLARONE™
Manuscripts

Health professionals' experiences and views on obstetric ultrasound in Vietnam: a regional, cross-sectional study

Sophia Holmlund[1*], Lan Pham Thi[2], Kristina Edvardsson[1,3], Phuc Ho Dang[4], Joseph Ntaganira[5], Rhonda Small[3,6], Hussein Lesio Kidanto[7], Matilda Ngarina[8], Ingrid Mogren[1,3]

[1]Department of Clinical Sciences, Obstetrics and Gynecology, Umeå University, Umeå, Sweden
[2]Department of Dermatology and Venereology, Hanoi Medical University, Hanoi, Vietnam
[3]Judith Lumley Centre, School of Nursing and Midwifery, La Trobe University, Melbourne, Australia
[4]Department of Probability and Mathematical Statistics, Institute of Mathematics, Vietnam Academy of Science and Technology, Hanoi, Vietnam
[5]School of Public Health, College of Medicine and Health Sciences, University of Rwanda, Kigali, Rwanda
[6]Department of Women's and Children's and Reproductive Health, Karolinska Institutet, Stockholm, Sweden
[7]Department of Obstetrics and Gynecology, Aga Khan University, Dar es Salaam, Tanzania
[8]Department of Obstetrics and Gynecology, Muhimbili National Hospital, Dar es Salaam, Tanzania

Email addresses:
SH: sofia.holmlund@umu.se
LPT: lanphamthi009@gmail.com
KE: k.edvardsson@latrobe.edu.au
PHD: hodang54@yahoo.com
JN: jntaganira@ur.ac.rw
RS: r.small@latrobe.edu.au
HLK: hkidanto@gmail.com
MN: mmatty71@gmail.com
IM: ingrid.mogren@umu.se

[*]Corresponding author

For peer review only

For peer review only - http://bmjopen.bmj.com/site/about/guidelines.xhtml

Address: Department of Clinical Sciences, Obstetrics and Gynecology, Umeå University, SE 901 87 Umeå, Sweden, sofia.holmlund@umu.se, +46 90-7850463

For peer review only

For peer review only - http://bmjopen.bmj.com/site/about/guidelines.xhtml

Abstract

Objectives

Obstetric ultrasound is an important part of antenatal care in Vietnam, although there are great differences in access to antenatal care and ultrasound services across the country. The aim of this study was to explore Vietnamese health professionals' experiences and views of obstetric ultrasound in relation to clinical management, resources and skills.

Design

A cross-sectional questionnaire study was performed as part of the **CRO**ss **C**ountry **U**ltra**S**ound study (CROCUS study).

Setting

Health facilities (N=29) in urban, semi-urban and rural areas of Hanoi region, Vietnam.

Participants

Participants were 289 obstetricians/gynaecologists and 535 midwives.

Results

A majority (88%) of participants agreed that "every woman should undergo ultrasound examination" in pregnancy to determine gestational age. Access to ultrasound at participants' workplaces was reported as good regardless of health facility level. Most participants

For peer review only

performing ultrasound reported high level skills for fetal heart rate examination (70%), whereas few (23%) reported being skilled in examination of the anatomy of the fetal heart. Insufficient ultrasound training leading to suboptimal pregnancy management was reported by 37% of all participants. “Better quality of ultrasound machines”, “more physicians trained in ultrasound” and “more training for health professionals currently performing ultrasound” were reported as ways to improve the utilisation of ultrasound.

Conclusions

Obstetric ultrasound is used as an integral part of antenatal care at all selected health facility levels in the region of Hanoi, and access was reported as high. However, reports of insufficient ultrasound training resulting in suboptimal pregnancy management indicates a need for additional training of ultrasound operators to improve utilisation of ultrasound.

Strengths and limitations of this study

- The study questionnaire was developed based on the results from earlier qualitative studies performed with obstetricians/physicians and midwives/nurses in six different countries (the CROCUS study).
- The strengths of this study include participants of different health professional categories recruited from different levels of the health care system in urban, semi-urban and rural areas of Hanoi.
- The research team comprised two Vietnamese researchers familiar with the setting and the health care system which strengthens the interpretation of data.

For peer review only

- One limitation of this study may be the translation of the questionnaire from English to Vietnamese, however, measures to reduce the risk of losing the intended meaning of questions and statements were implemented.
- Since previous studies within this research domain are lacking, the power calculation was based on assumptions of proportions for one outcome variable in relation to one background variable, and may therefore mean uncertainty of the required study sample.

Keywords

prenatal ultrasonography; health personnel; obstetricians; midwives; prenatal care; pregnancy; Vietnam

For peer review only

Background

Sufficient antenatal care (ANC) services and skilled birth attendance are important factors contributing to safer deliveries, reductions in obstetric complications, and decreased maternal and neonatal morbidity and mortality [1]. Timely and appropriate evidence-based practices in ANC including health promotion, screening and diagnosis, and prevention for diseases can save lives [2]. Since 2016, the World Health Organization (WHO) recommends eight ANC visits during pregnancy and one ultrasound examination before 24 weeks of gestation. The aim of the recommendation of an early ultrasound scan is to estimate gestational age, improve detection of multiple pregnancy and fetal anomaly, reduce induction of post-term pregnancy and improve women's pregnancy experience [3]. Ultrasound is an important part of ANC in high income countries [4], and clinical trials show that ultrasound may improve management and pregnancy outcomes in the developing world [5].

The utilisation of reproductive health services in Vietnam has substantially increased over recent decades, but there are still inequities in the country [6]. Low education, poverty, ethnic minority status [7] and living in rural areas are factors associated with decreased access to reproductive health services [6]. Ultrasound has become a central tool in ANC services in Vietnam [8]. Currently the Ministry of Health in Vietnam recommends at least four ANC visits [9], in accordance with the previous recommendation by WHO [10]. Additionally, three ultrasound examinations are recommended during pregnancy, in gestational week 11-13, 20-24 and 30-32 [11]. In 2014, more than 70% of pregnant women in Vietnam received at least four ANC visits [12]. However, almost half of these women reported not having urine or blood samples taken or blood pressure measurement done during their last pregnancy [12]. The private health sector is a continuously growing part of health care services in Vietnam, especially for provision of legal abortion services and obstetric ultrasound examinations [13]. Limited salary

For peer review only - http://bmjopen.bmj.com/site/about/guidelines.xhtml

for physicians creates incentives for both public and private practitioners to search for additional income through provision of these services [14 15]. Commercialisation of ultrasound services has led to urban Vietnamese women having an average of more than six ultrasound scans during pregnancy which is a high number of examinations from an international perspective [14].

Study rationale

Few studies have investigated the use of obstetric ultrasound in Vietnam from health professionals' perspectives, although ultrasound is frequently used during pregnancy. This study serves to fill a knowledge gap and can benefit authorities in their work to further develop education and improvement of guidelines on obstetric ultrasound use. This study is part of the **CRO**ss **C**ountry **U**ltra**S**ound study (CROCUS) investigating health professionals' experiences and views of the use of ultrasound in high-, middle and low-resource countries.

Aims

The overall aim of this study was to explore different aspects of obstetric ultrasound in Vietnam from health professionals' perspectives.

The research questions investigated were:

- What are health professionals' views of the role of obstetric ultrasound for clinical management of pregnancy?
- How do health professionals view access to obstetric ultrasound?
- How do health professionals assess their skills in performing obstetric ultrasound examinations?

For peer review only

- What do health professionals believe could improve the utilisation of obstetric ultrasound?

Materials and methods

The Vietnamese setting

Since 2010, Vietnam has been classified as a low to middle income country and has undergone substantial economic development in recent decades [1]. The maternal mortality rate has decreased from 139/100 000 in 1990 to 54/100 000 live births in 2015 [16]. Most inpatient health care is provided by public hospitals but for outpatient care, private clinics account for a large number of patients [17]. Vietnam is divided into 63 provinces, 698 districts and 11,121 communes [18]. In each commune, a village health worker (VHW) provides health promotion, immunisation, nutrition services and attends births in remote areas [19]. At the community health centre level (CHS), a midwife or an assistant doctor is in charge of maternal health services and provides ANC, assists normal delivery, and provides postnatal care, immunisation services and supervision of the VHW. At district level, ANC, delivery care including caesarean sections and newborn care are provided at hospitals [19] while maternity homes deliver basic prenatal and delivery services [17 19]. Provincial hospitals provide more specialised health care, and referrals from lower health care levels to provincial level are undertaken if complications occur during pregnancy or delivery [19]. At the top of the health care system there are several national hospitals providing specialised care and receiving referrals from lower levels [17].

Study design

For peer review only

This cross-sectional study used a questionnaire to investigate a number of research questions related to obstetric ultrasound with obstetricians/gynaecologists and midwives providing pregnancy, delivery and postpartum care to women in the region of Hanoi, Vietnam.

Sampling

Owing to the lack of findings from similar studies, a sample size of 290 obstetricians/gynaecologists and a corresponding number of midwives (n=290) was calculated based on plausible estimations of prevalence of background characteristics and outcome variables. The calculation was based on the outcome requiring the largest sample size "every woman should undergo ultrasound examination in pregnancy to determine gestational age" and the background variable "work experience over and under 5 years", to detect a difference in proportion of 0.10 with a power of 80% and a significance level of 5%.

Purposive sampling was used to obtain a representative sample of health professionals caring for pregnant women at different levels of health facilities in urban, semi-urban and rural areas in the region of Hanoi. One national hospital, one provincial hospital, 24 district hospitals and three maternity homes were included in the study, in total 29 health facilities.

Questionnaire

The study questionnaire was developed based on the results from the earlier qualitative studies performed in the CROCUS study [8 20-28]. Sociodemographic characteristics, evaluation of self-reported skills in performing ultrasound and questions about access to obstetric ultrasound and health professionals' views on what may improve utilisation of ultrasound in Vietnam were included, among other items. The questionnaire included both questions and statements, and the items had either fixed or Likert-scale response options. This analysis investigates the research questions noted above, utilising 45 of the 105 questions and

For peer review only - http://bmjopen.bmj.com/site/about/guidelines.xhtml

statements. The questionnaire was developed first in English and thereafter translated to Vietnamese by a native Vietnamese speaker independent of the research team. The Vietnamese version of the questionnaire was also back-translated to English by another external person. This check resulted in minor adjustments of some words, but demonstrated that the Vietnamese translation had retained the overall meaning of the English. The questionnaire was pilot-tested with 10 obstetricians, 6 midwives and 2 sonographers. No further revisions of the questionnaire were required as a result of piloting.

Data collection procedures including recruitment of study participants

The data collection was performed in April 2017 by four experienced data collectors supervised by two Vietnamese senior researchers in the research team. Before the start of the data collection, data collectors were trained by the research team and all questions and statements in the questionnaire were discussed to ensure correct understanding. The two Vietnamese researchers initiated contact with the directors of all selected health facilities and all of them agreed to assist with recruitment of participants. For this study, we aimed to include health professionals caring for pregnant women and with different experiences in relation to use of obstetric ultrasound. Eligible participants were health professionals managing pregnant women at the maternity wards on the day of data collection at each study site. No eligible participant declined participation in the study. The primary sample included 890 participants. Six individuals working as radiology technicians and 60 sonographers were excluded from the primary sample. The final sample (N=824) included the following health professionals: obstetricians/gynaecologists (n=289) and midwives (n=535). Participation was anonymous and all questionnaires were given a unique code. Safe storage of questionnaires was undertaken in accordance with national procedures and regulations. Data were entered

For peer review only

into an SPSS file at the Hanoi Medical University, by two experienced data clerks. To evaluate the quality of the data entry, every 10th questionnaire based on the number order of identification codes, was selected for data re-entry. The data from all 107 variables in 89 questionnaires were re-entered in the SPSS file by the first author. The rate of error was 1.4 %. The identified errors in the SPSS file were corrected.

Patient and Public Involvement

This research was done without patient involvement.

Independent variables

Age was calculated as a continuous variable using birth year and year of data collection. For some analyses age was dichotomised as age 34 years or less and 35 years and above. *Gender* included female or male. *Health profession* included the following response options in the questionnaire: obstetrician/gynaecologist, general practitioner, resident physician, physician other (please specify), midwife, radiologist/sonographer and “other” (please specify). *Health profession* was thereafter categorised into two groups: obstetricians/gynaecologists and midwives. Resident physicians undergoing post-graduate training (n=9) and general practitioners (n=12) were also included in the category obstetricians/gynaecologists because they worked at the same department and performed similar work tasks as the obstetricians/gynaecologists. One participant who was an anaesthesiologist by profession, but was working with maternity care, was categorised as an obstetrician/gynaecologist. One nurse working in maternity care was categorised as a midwife. *Health facilities* included the response options national hospital, provincial hospital, district hospital and maternity home. The variable *health facilities* was dichotomised into national hospital/provincial hospital and district hospital/maternity home in some analyses. *Area of health facility* was categorised as

For peer review only - http://bmjopen.bmj.com/site/about/guidelines.xhtml

hospitals in urban (n=7), semi-urban (n=5) and rural (n=17) areas of Hanoi. *Type of health care* was classified as public, private and both public and private health care. No participant reported working only in private health care. *Number of ultrasound examinations indicated in an uncomplicated pregnancy* was categorised based on the three recommended number of ultrasound examinations by the Ministry of Health in Vietnam; 3 examinations or less and 4 examinations or more.

Dependent variables

The dependent variables with fixed response alternatives are presented in Table 1. For the statements related to "the role of ultrasound for clinical management" and "resources and training of obstetric ultrasound", the response options were dichotomised into *disagree* or *strongly disagree* and *agree* or *strongly agree* in logistic regression analyses. The question "do you have a role in decision-making regarding clinical management on the basis of obstetric ultrasound examinations" was both used as an independent and dependent variable and the response options were dichotomised into *no* and *yes* for some analyses. The response options of the statements related to "improving utilisation of ultrasound" were categorised as *not at all* or *not very much* and *a fair amount* or *a great deal* in logistic regression analyses. The response option *neutral* or *don´t know* was not included in either of these categories. For most statements, the response options *neutral* and *don´t know* were selected by a small proportion of the participants.

Table 1. Questions or statements and their response options in the questionnaire

How often do you perform obstetric ultrasound examinations? [a]
How do you rate your skills in ultrasound in relation to the assessment/evaluation of:<br>• Fetal presentation[b]<br>• Localisation of the placenta[b]<br>• Fetal heart rate[b]<br>• Amniotic fluid amount[b]<br>• Gestational age estimated by CRL (crown-rump-length)[b]<br>• Gestational age estimated by biparietal diameter, femur length and abdominal diameter[b]

For peer review only

• Cervical length[b]<br>• Fetal heart: 4 chamber view[b]<br>• Fetal heart: aorta and pulmonary artery[b]<br>• Doppler: umbilical artery[b]
Do you have a role in decision-making regarding clinical management on the basis of obstetric ultrasound examinations?[c]
How often do you make decisions based on the results from obstetric ultrasound examinations in your clinical work? [a]
What do you believe would improve the utilisation of ultrasound at your clinic/work place?<br>• More ultrasound machines[d]<br>• Better quality of ultrasound machines[d]<br>• More training for health professionals currently performing ultrasound[d]<br>• More doctors trained in ultrasound[d]<br>• (More) midwives trained in ultrasound[d]
Statements on ultrasound resources and training<br>• Pregnant women in my country have access to dating ultrasound (i.e. estimation of gestational age)[e]<br>• Pregnant women in my country have access to fetal anomaly screening[e]<br>• Pregnant women in my country have access to obstetric ultrasound independent of area of living[e]<br>• Pregnant women in my country have access to obsteric ultrasound independent of income[e]<br>• There are enough resources in my country to provide *medically indicated* obstetric ultrasound examinations to pregnant women who need it[e]<br>• At my workplace, there is always access to obstetric ultrasound when it is needed[e]<br>• At my workplace, lack of ultrasound training of the ultrasound operator sometimes leads to suboptimal pregnancy management[e]<br>• Maternity care in my country would improve if midwives were qualified to perform basic ultrasound examinations[e]
Statements on the role of ultrasound in clinical management of pregnancy<br>• Ultrasound is decisive in pregnancy management[e]<br>• Every woman should undergo ultrasound examination in pregnancy to determine gestational age[e]<br>• It is irresponsible of a pregnant woman to decline a dating scan[e]<br>• Ultrasound is safe to use for the pregnant woman and the fetus irrespective of the number of examinations[e]<br>• Ultrasound is important for expectant parents to bond with their fetus during pregnancy[e]

[a]Response options: Never, On a daily basis, On a weekly basis, On a monthly basis, More seldom than on a monthly basis
[b]Response options: No skills, Skill-level low, Skill-level intermediate, Skill-level high
[c]Response options: No, Yes a minor role, Yes a moderate role, Yes a major role
[d]Response options: Not at all, Not very much, A fair amount, A great deal, Don't know
[e]Response options: Strongly agree, Agree, Neutral, Disagree, Strongly disagree

Statistical analysis

For categorical variables, frequencies and percentages were analysed and Pearson's Chi-Square test was used for test of difference, with the level of significance set at $p<0.05$. For continuous variables, mean values and their standard deviations (SD) were presented. Univariable and multivariable logistic regression was undertaken and are presented with odds ratios (OR) and their 95% confidence intervals (CI). The independent and dependent variables used for logistic regression are reported in their specific sections above as well as in Table 1. All independent variables were entered into the univariable logistic regression analysis,

For peer review only

For peer review only - http://bmjopen.bmj.com/site/about/guidelines.xhtml

however only the statistically significant variables were included in the final multivariable logistic regression models. Statistical analyses were performed using SPSS version 23.

Results

Background characteristics of the study sample

A total of 824 participants aged 21-60 years (mean age 34.8 years) were enrolled in the study. The distribution of health professionals was obstetricians/gynaecologists (35.1%), and midwives (64.9%; Table 2). One third of the participants (28.1%) was performing obstetric ultrasound and mainly on a daily basis (66.5%). All obstetricians/gynaecologists working in maternity homes (100%) were performing ultrasound as well as a majority of obstetricians/gynaecologists working in provincial hospitals (84.9%), district hospitals (75.2%) and national hospitals (75.7%). The mean estimated number of ultrasound examinations per day was 15.7 (range 1-100) for obstetricians/gynaecologists. Obstetricians/gynaecologists performing more than ten examinations per day were significantly older (≥35 years) than those obstetricians/gynaecologists performing ten or fewer examinations per day ($p<0.001$). A few participants at all health care levels (8.3%) reported that midwives performed ultrasound in their workplace. Participants at district hospitals (7.0%) and maternity homes (6.3%) reported the lowest percentage of midwives performing ultrasound in their workplace.

For peer review only

Table 2. Background characteristics of the study sample (N=824)

Variable	All health professionals<br>Total= 824<br>n (%)	Obstetricians/Gynaecologists<br>Total=289<br>n (%)	Midwives<br>Total=535<br>n (%)
**Age (years)**	***811 (98.4)***	***286 (99.0)***	***525 (98.1)***
Mean; SD[a]	34.8; 8.7	36.6; 9.2	33.7; 8.3
Min-Max	21-60	23-60	21-55
**Years in profession**	***818 (99.3)***	***288 (99.7)***	***530 (99.1)***
Mean; SD[a]	10.5; 8.3	10.4; 8.9	10.5; 8.1
Min-max	0-35	0-32	0.5-35
**Years in health care**	***817 (99.2)***	***287 (99.3)***	***530 (99.1)***
Mean; SD[a]	11.1; 8.5	11.6; 9.2	10.9; 8.2
Min-max	0-38	0-38	0.5-35
**Gender**	***824 (100)***	***289 (100)***	***535 (100)***
Male	123 (14.9)	123 (42.6)	0
Female	701 (85.1)	166 (57.4)	535 (100.0)
**Marital status**	***817 (99.2)***	***287 (99.3)***	***530 (99.1)***
Married	714 (87.4)	242 (84.3)	472 (89.1)
Separated/Divorced	1 (0.1)	0 (0.0)	1 (0.2)
Widowed	4 (0.5)	2 (0.7)	2 (0.4)
Not married/Single	98 (12.0)	43 (15.0)	55 (10.4)
**Having children**	***821 (99.6)***	***288 (99.7)***	***533 (99.6)***
Yes	684 (83.3)	230 (79.9)	454 (85.2)
No	137 (16.7)	58 (20.1)	79 (14.8)
**Type of health care**	***823 (99.9)***	***289 (100)***	***534 (99.8)***
Public	789 (95.9)	268 (92.7)	521 (97.6)
Both public and private	34 (4.1)	21 (7.3)	13 (2.4)
**Level of health facility[b]**	***824 (100)***	***289 (100)***	***535 (100)***
National hospital	144 (17.5)	74 (25.6)	70 (13.1)
Provincial hospital	184 (22.3)	86 (29.8)	98 (18.3)
District hospital	464 (56.3)	121 (41.9)	343 (64.1)
Maternity home	32 (3.9)	8 (2.8)	24 (4.5)
**Area of health facility[c]**	***824 (100)***	***289 (100)***	***535 (100)***
Urban	439 (53.3)	191 (66.1)	248 (46.4)
Semi-urban	129 (15.7)	35 (12.1)	94 (17.6)
Rural	256 (31.1)	63 (21.8)	193 (36.1)
**Provision of maternity services[d]**			
Antenatal care	683 (83.0)	261 (90.3)	422 (79.0)
Intrapartum care	642 (78.0)	245 (84.8)	397 (74.3)
Postpartum care	688 (83.6)	235 (81.3)	453 (84.8)
Do not currently provide maternity care	32 (3.9)	10 (3.5)	23 (4.3)
**Performing ultrasound[e]**	***823 (99.9)***	***289 (100)***	***534 (99.8)***
Yes	231 (28.1)	228[f] (78.9)	3 (0.6)
No	592 (71.9)	61 (21.1)	531 (99.4)

[a]SD= Standard Deviation
[b]Number of participants at specified health facilities
[c]Number of participants at specified areas of health facilities
[d]Item in questionnaire: "Which of the following maternity services do you provide? (Please tick all that apply)"
[e]Performing obstetric ultrasound examinations
[f]One participant have not rated the skills in relation to different tasks during ultrasound examinations

For peer review only

The role of obstetric ultrasound

Most participants (66.2%) agreed or strongly agreed that "ultrasound is decisive in pregnancy management". Obstetricians/gynaecologists reported significantly lower agreement (55.7%) with the statement "ultrasound is decisive in pregnancy management" than midwives (71.8%; $p<0.001$). A majority (87.5%) of participants, independent of health profession, agreed or strongly agreed that "every woman should undergo ultrasound examination to determine gestational age". Most participants (75.0%) agreed or strongly agreed that "it is irresponsible of a pregnant woman to decline a dating ultrasound" and there was no significant difference in opinion between different health professionals. There was a significant difference in opinion between the different health professionals for the statement "ultrasound is safe to use for the woman and the fetus irrespective of the number of examinations", where obstetricians/gynaecologists were more likely to agree or strongly agree than midwives (OR 1.96; 95% CI 1.22-3.17). Participants reported a mean number of 5.9 ultrasound examinations to be medically indicated during an uncomplicated pregnancy (obstetricians/gynaecologists: SD 2.7, range 2-15, and midwives: SD 2.6, range 2-20). Midwives in comparison to obstetricians/gynaecologists, were more likely to agree or strongly agree that "ultrasound is important for expectant parents to bond with their fetus during pregnancy" (OR 1.59; 95% CI 1.13-2.26). The assessment that four ultrasound examinations or more are medically indicated in an uncomplicated pregnancy was associated with higher agreement with the statement "ultrasound is important for expectant parents to bond with their fetus during pregnancy", compared to those assessing that three ultrasound examinations or fewer are medically indicated in a normal pregnancy (OR 1.61; 95% CI 1.03-2.50, adjusted for health profession and performing ultrasound or not).

For peer review only

Access to obstetric ultrasound

Most of the participants (95.6%-100%), regardless of health facility level, agreed or strongly agreed with the statement "there is always access to obstetric ultrasound when needed at my workplace". Almost all participants (95.4%-100%) at all health facility levels reported that they agreed or strongly agreed with the statement "pregnant women in the country have access to dating ultrasound". A majority of participants at all health facility levels (93.8%-95.1%), agreed or strongly agreed that "pregnant women in the country have access to fetal anomaly screening". Midwives were more likely to agree or strongly agree that "pregnant women in the country have access to ultrasound independent of area of residence, compared with obstetricians/gynaecologists (OR 2.54; 95% CI 1.60-4.02). Participants in national hospitals and provincial hospitals reported significantly lower agreement with the statement "pregnant women in my country have access to ultrasound independent of income" than participants in district hospitals and maternity homes (p<0.001). Further results are presented in Table 3.

Table 3. Health professionals' views on specified statements[a] (N=824)

**Statement**	**Obstetricians/ Gynaecologists** Total=289[c] **n (%)**	**Midwives** Total=535[c] **n (%)**	**p-value**[b]
**Pregnant women in my country have access to dating ultrasound (i.e. estimation of gestational age)**	***279 (96.5)***	***521 (97.4)***	0.98
Agree/Strongly agree	275 (98.6)	512 (98.3)	
Disagree/Strongly disagree	4 (1.4)	9 (1.7)	
**Pregnant women in my country have access to fetal anomaly screening**	***276 (95.5)***	***514 (96.1)***	0.16
Agree/Strongly agree	269 (97.5)	509 (99.0)	
Disagree/Strongly disagree	7 (2.5)	5 (1.0)	
**Pregnant women in my country have access to obstetric ultrasound independent of area of residence**	***248 (85.8)***	***485 (90.7)***	<0.001
Agree/Strongly agree	203 (81.9)	446 (92.0)	
Disagree/Strongly disagree	45 (18.1)	39 (8.0)	
**Pregnant women in my country have access to obstetric ultrasound independent of income**	***228 (78.9)***	***475 (88.8)***	0.082
Agree/Strongly agree	178 (78.1)	398 (83.8)	
Disagree/Strongly disagree	50 (21.9)	77 (16.2)	

For peer review only

**There are enough resources in my country to provide medically indicated obstetric ultrasound examinations to pregnant women who need it**	***234 (81.0)***	***447 (83.6)***	0.002
Agree/Strongly agree	185 (79.1)	395 (88.4)	
Disagree/Strongly disagree	49 (20.9)	52 (11.6)	
**At my workplace, there is always access to obstetric ultrasound when it is needed**	***282 (97.6)***	***519 (97.0)***	0.010
Agree/Strongly agree	272 (96.5)	515 (99.2)	
Disagree/Strongly disagree	10 (3.5)	4 (0.8)	
**At my workplace, lack of ultrasound training of the ultrasound operator sometimes leads to suboptimal pregnancy management**	***237 (82.0)***	***452 (84.5)***	0.086
Agree/Strongly agree	115 (48.5)	187 (41.4)	
Disagree/Strongly disagree	122 (51.5)	265 (58.6)	

[a]The response option *neutral* was excluded from analyses
[b]Pearsons Chi-Square Test with Yates' continuity correction for test of differences in proportion between the two groups of obstetricians/gynaecologists and midwives
[c]The number of participants included in each analysis are presented in relation to the total sample of each group of obstetricians/gynaecologists and midwives

Ultrasound operators' decision-making and self-rated skills

Almost all obstetricians/gynaecologists (92.2%) and a majority of midwives (59.4%) reported having a role in decision-making regarding clinical management on the basis of obstetric ultrasound examinations. There was a significant difference in the proportion of obstetricians/gynaecologists (38.6%) and midwives (20.5%) reporting having a major role in decision-making compared to having a minor or moderate role in decision-making ($p<0.001$). Approximately one third of all health professionals (37.1%) reported that they agreed or strongly agreed with the statement "at my workplace, lack of ultrasound training of the ultrasound operator sometimes leads to suboptimal pregnancy management". Health professionals performing ultrasound were asked to rate their skills in relation to different tasks during ultrasound examinations (Figure 1). For all the obstetricians/gynaecologists performing ultrasound (n=228), fetal heart rate was the examination where most participants reported high skills (70.0%) and examination of the fetal heart: aorta and pulmonary artery was the examination with the lowest proportion of self-rated high skill level (22.5%).

Figure 1.

For peer review only

Improving utilisation of obstetric ultrasound

When health professionals were asked to assess how much particular strategies could improve the utilisation of ultrasound, “better quality of ultrasound machines” (94.0%), “more training for health professionals currently performing ultrasound” (92.8%) and “more physicians trained in ultrasound” (93.2%) were the statements with the highest numbers of participants reporting a fair amount or a great deal. Obstetricians/gynaecologists were more likely to agree that “more training for health professionals currently performing ultrasound would improve the utilisation of ultrasound” compared to midwives (OR 2.60; 95% CI 1.24-5.46). Further results are presented in Table 4. A majority of participants (69.0%) agreed or strongly agreed with the statement “maternity care in my country would improve if midwives were qualified to perform basic ultrasound examinations”, however midwives were more likely to agree or strongly agree than obstetricians/gynaecologists (OR 5.09; 95% CI 3.41-7.61). Participants working in rural hospitals (79.6%) and semi-urban hospitals (78.3%) were also more likely to agree with the previous statement than participants working in urban hospitals (60.0%) ($p<0.001$).

Table 4. Health professionals’ views on factors that may improve utilisation of obstetric ultrasound, presented through preformed statements

Discussion

The main findings of this study are that access to obstetric ultrasound was generally reported as satisfactory regardless of participants’ health profession and health facility level. Obstetricians/gynaecologists reported high self-rated skill levels for most obstetric ultrasound examinations, although one third of all health professionals reported that “lack of ultrasound training sometimes leads to suboptimal pregnancy management”. “Better quality of

For peer review only - http://bmjopen.bmj.com/site/about/guidelines.xhtml

ultrasound machines", "more training for health professionals' currently performing ultrasound" and "more physicians trained in ultrasound" were reported as factors that could improve the utilisation of ultrasound to the greatest extent. For participants at health facilities in rural and semi-urban areas compared to those in urban areas, the great majority reported that "maternity care in my country would improve if midwives were qualified to perform basic ultrasound examinations".

The role of obstetric ultrasound

Obstetric ultrasound is one of the most important technological advances in pregnancy surveillance [29]. The majority of health professionals in our study agreed or strongly agreed with the statement "ultrasound is decisive in pregnancy management" but unexpectedly one third of our participants were neutral or disagreed with this statement. Obstetricians/gynaecologists who mainly performed ultrasound reported lower agreement with the above statement than midwives who mainly did not perform ultrasound examinations. CROCUS-study findings in Rwanda also demonstrated that health professionals not themselves performing ultrasounds have more liberal attitudes towards ultrasound use then health professionals performing these examinations [30]. Health professionals sometimes fear that "routinisation" of ultrasound as an unquestioned and integral part of pregnancy management can exert a negative impact on pregnant women's informed consent, and that its increasing use for fetal examination may have consequences for how disability is viewed in society [26 27 31]. The use of ultrasound may also reduce attention on important clinical parameters such as measurement of blood pressure and proteinuria [25 32]. Previous research from Vietnam has shown that many ultrasound examinations are performed just for reassurance of fetal wellbeing [14]. Although problems of access to obstetric ultrasound exist in many low-resource countries, inappropriate use of ultrasound examinations with no

For peer review only

effect on pregnancy outcomes still occurs. In a study from Uganda, more than half of all ultrasound examinations performed were classified as inappropriate, for example dating of pregnancy in a suboptimal gestational week or requesting an ultrasound without medical indication [33]. The Ministry of Health in Vietnam recommends three ultrasound examinations including one examination also in the third trimester. However, a routine late pregnancy ultrasound in unselected or low-risk populations has been reported to have no benefit for mother or baby [34]. It is known that Vietnamese women generally undergo many ultrasound examinations during pregnancy [14], and health professionals performing ultrasound in our study also reported performing up to 100 scans per day per ultrasound operator. High workload can lead to inadequate provision of information to women by the physician, including about indication for ultrasound and the results of the ultrasound examination [14 35]. In a broader perspective, non-medical ultrasound examinations during pregnancy consume resources unnecessarily, with negative impact on other maternal health care services. Issuing medical guidelines stating clear indications for ultrasound surveillance during pregnancy are therefore important and may contribute to more appropriate allocation of resources within the health care system in Vietnam.

Access to obstetric ultrasound

Health professionals in our study generally reported satisfactory access to obstetric ultrasound when needed in their own workplace. Access to ultrasound has increased significantly in many resource-limited settings although there are still large differences in access within and between countries [36]. Our study was performed in the area around Hanoi, i.e. the capital of Vietnam, and it is plausible to believe that access to obstetric ultrasound is higher in this region than in other parts of Vietnam. In Hanoi, ultrasound scans are easily accessible and affordable for most pregnant women both within the public and private health care systems.

For peer review only - http://bmjopen.bmj.com/site/about/guidelines.xhtml

Increased ANC attendance and health facility delivery rates have been seen in Vietnam in recent years, but also increased inequities in maternity care utilisation [37 38], primarily among women with multiple socioeconomic vulnerabilities [37].

Ultrasound operators' skills

Participants performing ultrasound in our study reported high or intermediate skill levels for the majority of the specified ultrasound examinations. Proper training of health professionals performing ultrasound is critically important [29] especially since ultrasound is operator dependent to a large extent [5]. Further, ultrasound training should include ethics, discuss use and misuse of the ultrasound tool in addition to the quality of ultrasound performance and clinical implications of its use [29], to ensure maximum diagnostic utility and high levels of sensitivity and specificity [5].

In our study, one third of all health professionals believed that insufficient training of the ultrasound operator sometimes leads to suboptimal pregnancy management. Another study from Vietnam reports that physicians experience less knowledge about fetal anomalies, lack advanced training and do not have appropriate equipment and professional protocols that could support their practice in performing obstetric ultrasound [39]. Inadequately skilled health professionals may cause inadvertent harm, for example, by providing false-positive diagnosis where termination of pregnancy may be an option. Alternatively, false-negative information because of inadequate ability to recognise the signs of important diagnoses can result in parents not being offered further investigations and being inappropriately reassured that everything is normal [40].

Improving utilisation of ultrasound

For peer review only - http://bmjopen.bmj.com/site/about/guidelines.xhtml

Our results indicate an increased number of physicians to become appropriately educated in ultrasound examinations, regular in-service training sessions and better quality of ultrasound equipment. Lack of training of health care providers has been seen as the most common barrier to regular ultrasound use, although lack of equipment and maintenance and costs for machines also are explanatory factors [41]. A review of ultrasound training in low and middle income countries (LMICs) shows that health professionals often do not meet the WHO criteria in relation to number of scans, supervision and length of training [42]. The obstacles to ultrasound training in LMICs include lack of time for training because of limited possibilities for absence from the workplace, and the logistics to access qualified teachers [42]. Our results also indicate that health professionals in Vietnam seem to have a very substantial workload. It has been shown that training of midwives in basic obstetric ultrasound may significantly improve pregnancy management [43-45]. In our study, the majority of midwives were positive to the idea of training midwives in ultrasound to improve utilisation of ultrasound. For rural and semi-urban hospitals, a majority of all participants were also positive to ultrasound-trained midwives. However, obstetricians/gynaecologists did not support the idea of training midwives in ultrasound as much as participating midwives. As in much of Vietnamese society, health professions are organised hierarchically. Nurses' roles can be seen as primarily carrying out doctors' orders, not to take their own initiatives [46 47], and it may be a plausible explanation for why physicians defend performing ultrasound as their duty. Shortages of nurses and midwives and lack of quality of training [48 49], may also be additional explanations for physicians not supporting the idea of training midwives in obstetric ultrasound.

Strengths and limitations

The strengths of this study include participants recruited from different levels of the health care system in urban, semi-urban and rural areas of Hanoi. We believe that the wide range of

For peer review only

health facilities is likely to be representative for the Vietnamese health care system. The research team comprised two Vietnamese researchers familiar with the setting and the health care system which strengthens the interpretation of data. An additional strength was that four experienced Vietnamese data collectors, familiar with the setting, collected all data. One limitation of this study may be the translation of the questionnaire from English to Vietnamese, with the risk of losing the intended meaning of questions and statements. However, measures to reduce this risk were implemented, including back-translation of the questionnaire. Another limitation might be the unequal distribution between the categories of physicians and midwives in this sample. Although we aimed for equal numbers of each category of health professional, as assessed in the power calculation, all eligible physicians and midwives working the day of data collection were included in the study. This resulted in a higher number of midwives. Since there is a lack of previous studies within this research domain, the power calculation was based on assumptions of proportions for one outcome variable in relation to one background variable. The assumptions in the power calculations therefore mean uncertainty of the required study sample. However, the results indicate that the sample size was probably adequate for investigating our questions of interest. There was no question in the questionnaire about whether the participant had received any formal ultrasound training and this may be considered a limitation. In Vietnam, it is widely recognised that participants in studies receive payment. All participants were therefore paid with a small sum for answering the questionnaire which may theoretically have affected the willingness of participation in the study.

For peer review only

Conclusions

Obstetric ultrasound is used as an integral part of antenatal care at all selected health facility levels in the region of Hanoi and access to obstetric ultrasound was also reported to be good

For peer review only - http://bmjopen.bmj.com/site/about/guidelines.xhtml

at all levels. Overall, participants performing obstetric ultrasound reported satisfactory self-rated skill levels. However, report of insufficient ultrasound training resulting in suboptimal pregnancy management in combination with the suggestion for more training to improve utilisation of ultrasound, indicates a need for additional in-service training of ultrasound operators. The proposal of educating midwives in obstetric ultrasound needs to be further evaluated.

Acknowledgements

Our sincere thanks to the participating health professionals for sharing their time and experiences and for the support by the heads of the Departments of Obstetrics and Gynaecology at the selected health facilities. Thanks go to the data collectors Do Nam Khanh, Nguyen Huyen Tram, Le Vu Thuy Huong and Nguyen Thi Hue for excellent performance of the data collection. We also thank Annika Åhman, Uppsala University, Sweden, for valuable contributions to the questionnaire and Gabriel Granåsen, Umeå University, Sweden for statistical support. We acknowledge the support received from Hanoi Medical University, Hanoi, Vietnam and Umeå University, Umeå, Sweden.

Abbreviations

CROCUS: Cross Country Ultrasound study; ANC: Antenatal care; WHO: World Health Organization; VHW: Village health worker; CHS: Community health centre level; SD: Standard deviation; OR: Odds ratio; CI: Confidence intervals; LMICs: Low and middle income countries.

Footnotes

For peer review only

For peer review only - http://bmjopen.bmj.com/site/about/guidelines.xhtml

Authors' contributions

IM is principal investigator of the CROCUS study. SH, LPT, KE, JN, RS, HLK, MN and IM designed the study. SH, KE and IM were the main responsible for developing the questionnaire with input from all co-authors. LPT, PHD, SH, KE and IM organised and performed the data collection together with data collectors. SH conducted the analyses in collaboration with IM, LPT and PHD. SH drafted the primary manuscript in collaboration with IM. All authors have contributed to and approved the final manuscript, tables and figures.

Funding

This research was made possible by grants from Umeå University, Västerbotten County Council, The Swedish Research Council, Sweden (2014-2672), the Swedish Research Council for Health, Working Life and Welfare (Forte) and the European Commission under a COFAS Marie Curie Fellowship (2013-2699).

Competing interests

The authors declare that they have no competing interests.

Ethics approval and consent to participate

Ethical approval was obtained from the Hanoi Medical University Review Board in Bio-Medical Research, reference 141/HMU IRB. The participants received both written and verbal information about the study including that participation was voluntary. All participants signed a consent form before completing the questionnaire.

For peer review only

Data sharing statement

The datasets used and/or analysed during the current study are available from the corresponding author on reasonable request.

For peer review only

For peer review only - http://bmjopen.bmj.com/site/about/guidelines.xhtml

References

1. Ministry of Health, Viet Nam, Partnership for Maternal, Newborn & Child Health, WHO, World Bank and Alliance for Health Policy and Systems Research. Success factors for women´s and children´s health: Viet Nam. Geneva: World Health Organization, 2014.
2. World Health Organization. WHO recommendations on antenatal care for a positive pregnancy experience, 2016.
3. Tuncalp, Pena-Rosas JP, Lawrie T, et al. WHO recommendations on antenatal care for a positive pregnancy experience-going beyond survival. *Bjog* 2017;124(6):860-62. doi: 10.1111/1471-0528.14599
4. L.J. Salomon, Z. Alfirevic, V. Berghella, C. Bilardo, E. Hernandez-Andrade, S. L. et.al. on behalf of the ISUOG Clinical Standards Committe. Practice guidelines for performence of the routine mid-trimester fetal ultrasound scan. *Ultrasound Obstet Gynecol (2010)* doi: 10.1002/uog.8831
5. Stanton K, Mwanri L. Global Maternal and Child Health Outcomes: The Role of Obstetric Ultrasound in Low Resource Settings. *World Journal of Preventive Medicine* 2013;1(3):22-29.
6. Duc NHC, Nakamura K, Kizuki M, et al. Trends in inequalities in utilization of reproductive health services from 2000 to 2011 in Vietnam. *Journal of Rural Medicine: JRM* 2015;10(2):65-78. doi: 10.2185/jrm.2902
7. Goland E, Hoa DT, Malqvist M. Inequity in maternal health care utilization in Vietnam. *International journal for equity in health* 2012;11:24. doi: 10.1186/1475-9276-11-24
8. Edvardsson K, Graner S, Thi LP, et al. 'Women think pregnancy management means obstetric ultrasound': Vietnamese obstetricians' views on the use of ultrasound during pregnancy. *Glob Health Action* 2015;8(1):28405. doi: 10.3402/gha.v8.28405
9. Ministry of Health, Viet Nam. Circular on Promulgation of Health Statistics Indicators at Commune, District, and Provincial Levels. (32/2014/TT-BYT), 2014.
10. WHO antenatal care randomized trial: manual for the implementation of the new model. Geneva: World Health Organization, 2002.
11. Ministry of Health, Viet Nam. National clinical guidelines on reproductive health care services. Ha Noi, 2009.
12. General Statitics Office and UNICEF. Viet Nam Multiple Indiciator Cluster Survey 2014, Final Report. Ha Noi, Viet Nam, 2015.
13. UNFPA. Improvning the Quality of Reproductive Health Care Services in Viet Nam. The Role of National Standards and Guidelines for Reproductive Health Care Services. Ha Noi, 2007.
14. Gammeltoft T, Nguyen HT. The commodification of obstetric ultrasound scanning in Hanoi, Viet Nam. *Reprod Health Matters* 2007;15(29):163-71. doi: 10.1016/S0968-8080(06)29280-2
15. Vujicic M, Shengelia B, Alfano M, et al. Physician shortages in rural Vietnam: using a labor market approach to inform policy. *Soc Sci Med* 2011;73(7):970-7. doi: 10.1016/j.socscimed.2011.06.010
16. The World Bank, Vietnam. http://data.worldbank.org, 2017.
17. WHO and the Ministry of Health Viet Nam. Health Service Delivery Profile Viet Nam, 2012.
18. Ministry of Health, Viet Nam. Dept. of Planning and Finance and Health Insurance. Social health insurance scheme in Vietnam. Achievements & Challanges. Tokyo, 2016.

For peer review only - http://bmjopen.bmj.com/site/about/guidelines.xhtml

19. Raven JL, X. Hu, D. Zhu, W. Dinh, TPH. et. al. Improving neonatal health in remote rural areas in China and Vietnam: synthesis report. *Liverpool School of Tropical Medicine, Liverpool UK,* 2015.

20. Ahman A, Kidanto HL, Ngarina M, et al. 'Essential but not always available when needed' - an interview study of physicians' experiences and views regarding use of obstetric ultrasound in Tanzania. *Glob Health Action* 2016;9(1):31062. doi: 10.3402/gha.v9.31062

21. Edvardsson K, Ntaganira J, Ahman A, et al. Physicians' experiences and views on the role of obstetric ultrasound in rural and urban Rwanda: a qualitative study. *Trop Med Int Health* 2016;21(7):895-906. doi: 10.1111/tmi.12718

22. Holmlund S, Ntaganira J, Edvardsson K, et al. Improved maternity care if midwives learn to perform ultrasound: a qualitative study of Rwandan midwives' experiences and views of obstetric ultrasound. *Glob Health Action* 2017;10(1):1350451. doi: 10.1080/16549716.2017.1350451

23. Åhman A, Edvardsson K, Lesio Kidanto H, et al. 'Without ultrasound you can't reach the best decision' – Midwives' experiences and views of the role of ultrasound in maternity care in Dar Es Salaam, Tanzania. *Sexual & Reproductive Healthcare* 2018;15:28-34. doi: https://doi.org/10.1016/j.srhc.2017.11.007

24. Edvardsson K, Small R, Persson M, et al. 'Ultrasound is an invaluable third eye, but it can't see everything': a qualitative study with obstetricians in Australia. *BMC Pregnancy Childbirth* 2014;14:363. doi: 10.1186/1471-2393-14-363

25. Ahman A, Persson M, Edvardsson K, et al. Two sides of the same coin-an interview study of Swedish obstetricians' experiences using ultrasound in pregnancy management. *BMC Pregnancy Childbirth* 2015;15:304. doi: 10.1186/s12884-015-0743-5

26. Edvardsson K, Mogren I, Lalos A, et al. A routine tool with far-reaching influence: Australian midwives' views on the use of ultrasound during pregnancy. *BMC Pregnancy Childbirth* 2015;15:195. doi: 10.1186/s12884-015-0632-y

27. Edvardsson K, Lalos A, Ahman A, et al. Increasing possibilities - Increasing dilemmas: A qualitative study of Swedish midwives' experiences of ultrasound use in pregnancy. *Midwifery* 2016;42:46-53. doi: 10.1016/j.midw.2016.09.009

28. Edvardsson K, Small R, Lalos A, et al. Ultrasound's 'window on the womb' brings ethical challenges for balancing maternal and fetal health interests: obstetricians' experiences in Australia. *BMC Med Ethics* 2015;16:31. doi: 10.1186/s12910-015-0023-y

29. Kongnyuy EJ, van den Broek N. The use of ultrasonography in obstetrics in developing countries. *Trop Doct* 2007;37(2):70-2. doi: 10.1177/004947550703700203

30. Holmlund S, Ntaganira J, Edvardsson K, et al. Health professionals' experiences and views on obstetric ultrasound in Rwanda: A cross-sectional study. *PLOS ONE* 2018;13(12):e0208387. doi: 10.1371/journal.pone.0208387

31. Edvardsson K, Åhman A, Fagerli TA, et al. Norwegian obstetricians' experiences of the use of ultrasound in pregnancy management. A qualitative study. *Sexual & Reproductive Healthcare* 2018;15:69-76. doi: https://doi.org/10.1016/j.srhc.2017.12.001

32. Edvardsson K, Graner S, Thi LP, et al. 'Women think pregnancy management means obstetric ultrasound': Vietnamese obstetricians' views on the use of ultrasound during pregnancy. *Glob Health Action* 2015;8:28405. doi: 10.3402/gha.v8.28405

33. Gonzaga MA, Kiguli-Malwadde E, Businge F, et al. Utilisation of obstetric sonography at a peri-urban health centre in Uganda. *The Pan African Medical Journal* 2010;7:24.

34. Bricker L, Medley N, Pratt JJ. Routine ultrasound in late pregnancy (after 24 weeks' gestation). *Cochrane Database of Systematic Reviews* 2015(6) doi: 10.1002/14651858.CD001451.pub4

35. Mensah YB, Nkyekyer K, Mensah K. The Ghanaian Woman's Experience and Perception of Ultrasound Use in Antenatal Care. *Ghana Medical Journal* 2014;48(1):31-38.

For peer review only - http://bmjopen.bmj.com/site/about/guidelines.xhtml

36. Sippel S, Muruganandan K, Levine A, et al. Review article: Use of ultrasound in the developing world. *International Journal of Emergency Medicine* 2011;4:72-72. doi: 10.1186/1865-1380-4-72
37. Van Minh H, Oh J, Giang KB, et al. Multiple vulnerabilities and maternal healthcare in Vietnam: findings from the Multiple Indicator Cluster Surveys, 2000, 2006, and 2011. *Global Health Action* 2016;9:10.3402/gha.v9.29386. doi: 10.3402/gha.v9.29386
38. Malqvist M, Lincetto O, Du NH, et al. Maternal health care utilization in Viet Nam: increasing ethnic inequity. *Bull World Health Organ* 2013;91(4):254-61. doi: 10.2471/BLT.12.112425
39. Gammeltoft T, Nguyen HT. Fetal conditions and fatal decisions: ethical dilemmas in ultrasound screening in Vietnam. *Soc Sci Med* 2007;64(11):2248-59. doi: 10.1016/j.socscimed.2007.02.015
40. Howe D. Ethics of prenatal ultrasound. *Best Practice & Research Clinical Obstetrics & Gynaecology* 2014;28(3):443-51. doi: https://doi.org/10.1016/j.bpobgyn.2013.10.005
41. Shah S, Bellows BA, Adedipe AA, et al. Perceived barriers in the use of ultrasound in developing countries. *Critical Ultrasound Journal* 2015;7(1):11. doi: 10.1186/s13089-015-0028-2
42. LaGrone LN, Sadasivam V, Kushner AL, et al. A review of training opportunities for ultrasonography in low and middle income countries. *Trop Med Int Health* 2012;17(7):808-19. doi: 10.1111/j.1365-3156.2012.03014.x
43. Swanson JO, Kawooya MG, Swanson DL, et al. The diagnostic impact of limited, screening obstetric ultrasound when performed by midwives in rural Uganda. *J Perinatol* 2014;34(7):508-12. doi: 10.1038/jp.2014.54
44. Nathan RO, Swanson JO, Swanson DL, et al. Evaluation of Focused Obstetric Ultrasound Examinations by Health Care Personnel in the Democratic Republic of Congo, Guatemala, Kenya, Pakistan, and Zambia. *Current Problems in Diagnostic Radiology* 2017;46(3):210-15. doi: https://doi.org/10.1067/j.cpradiol.2016.11.001
45. Bentley S, Hexom B, Nelson BP. Evaluation of an Obstetric Ultrasound Curriculum for Midwives in Liberia. *J Ultrasound Med* 2015;34(9):1563-8. doi: 10.7863/ultra.15.14.08017
46. Nancy Waxler-Morrison JMA, Elisabeth Richardson, Natalie A. Chambers. Cross-Cultural Caring: A handbook for Health Professionals. 2nd ed. Vancouver, Canada: UBC Press 2011.
47. Jones PS, O'Toole MT, Hoa N, et al. Empowerment of nursing as a socially significant profession in Vietnam. *Journal of Nursing Scholarship* 2000;32(3):317-21. doi: 10.1111/j.1547-5069.2000.00317.x
48. The World Bank. World Health Organization's Global Health Workforce Statistics, OECD, supplemented by country data 2013 [cited 2017 2017-01.15]. Available from: www.data.worldbank.org
49. World Health Organization, Western Pacific Region. Human resources for health. Action Framework for the Western Pacific region (2011-2015). Geneva, Switzerland, 2012.

For peer review only - http://bmjopen.bmj.com/site/about/guidelines.xhtml

765 # Figure legend

766

767 **Fig. 1. Obstetricians/Gynaecologists' self-rated skills for specified ultrasound**
768 **examinations (n=227).** Reported skill levels are presented with proportions (%)

769

For peer review only

For peer review only - http://bmjopen.bmj.com/site/about/guidelines.xhtml

Table 4. Health professionals' views on factors that may improve utilisation of obstetric ultrasound, presented through preformed statements

**Variable**	**More ultrasound machines**[a]			**Better quality of ultrasound machines**[a]		
	Not at all or Not very much[b] **n (%)**	A fair amount or A great deal[b] **n (%)**	p-value[c]	Not at all or Not very much[b] **n (%)**	A fair amount or A great deal[b] **n (%)**	p-value[c]
**Health profession**	***149 (20.1)***	***593 (79.9)***	0.042	***40 (5.2)***	***732 (94.8)***	0.036
Obstetricians/Gynaecologists	65 (24.3)	203 (75.7)		8 (2.8)	276 (97.2)	
Midwives	84 (17.7)	390 (82.3)		32 (6.6)	456 (93.4)	
**Level of health facility**	***149 (20.1)***	***593 (79.9)***	0.003	***40 (5.2)***	***732 (94.8)***	0.232
National hospital	31 (24.4)	96 (75.6)		3 (2.2)	135 (97.8)	
Provincial hospital	43 (28.3)	109 (71.7)		12 (7.2)	154 (92.8)	
District hospital	73 (16.9)	359 (83.1)		24 (5.5)	413 (94.5)	
Maternity home	2 (6.5)	29 (93.5)		1 (3.2)	30 (96.8)	
**Performing ultrasound**[d]	***149 (20.1)***	***592 (79.9)***	0.045	***40 (5.2)***	***731 (94.8)***	0.060
Yes	53 (25.0)	159 (75.0)		6 (2.6)	221 (97.4)	
No	96 (18.1)	433 (81.9)		34 (6.3)	510 (93.8)	
	**More training for health professionals currently performing ultrasound**[a]			**More physicians trained in ultrasound**[a]		
	Not at all or Not very much[b] **n (%)**	A fair amount or A great deal[b] **n (%)**	p-value[c]	Not at all or Not very much[b] **n (%)**	A fair amount or A great deal[b] **n (%)**	p-value[c]
**Health profession**	***48 (6.3)***	***717 (93.7)***	0.014	***43 (5.7)***	***714 (94.3)***	1.000
Obstetricians/Gynaecologists	9 (3.2)	269 (96.8)		16 (5.7)	263 (94.3)	
Midwives	39 (8.0)	448 (92.0)		27 (5.6)	451 (94.4)	
**Level of health facility**	***48 (6.3)***	***717 (93.7)***	0.097	***43 (5.7)***	***714 (94.3)***	0.047
National hospital	6 (4.5)	126 (95.5)		3 (2.3)	129 (97.7)	
Provincial hospital	15 (9.1)	149 (90.9)		15 (9.2)	148 (90.8)	
District hospital	23 (5.2)	416 (94.8)		22 (5.1)	410 (94.9)	
Maternity home	4 (13.3)	26 (86.7)		3 (10.0)	27 (90.0)	
**Performing ultrasound**[d]	***48 (6.3)***	***716 (93.7)***	0.034	***43 (5.7)***	***713 (94.3)***	0.950
Yes	7 (3.2)	215 (96.8)		12 (5.4)	211 (94.6)	
No	41 (7.6)	501 (92.4)		31 (5.8)	502 (94.2)	
	**(More) midwives trained in ultrasound**[a]					

For peer review only

	Not at all or Not very much[b] n (%)	A fair amount or A great deal[b] n (%)	p-value[c]
**Health profession**	***277 (37.5)***	***462 (62.5)***	<0.001
Obstetricians/Gynaecologists	165 (60.7)	107 (39.3)	
Midwives	112 (24.0)	355 (76.0)	
**Level of health facility**	***277 (37.5)***	***462 (62.5)***	0.003
National hospital	50 (38.2)	81 (61.8)	
Provincial hospital	79 (49.4)	81 (50.6)	
District hospital	139 (33.4)	277 (66.6)	
Maternity home	9 (28.1)	23 (71.9)	
**Performing ultrasound**[d]	***277 (37.5)***	***461 (62.5)***	<0.001
Yes	135 (62.2)	82 (37.8)	
No	142 (27.3)	379 (72.7)	

[a]Item in questionnaire: "What do you believe would improve the utilisation of ultrasound at your clinic/work place?"
[b]The response option *don´t know* was excluded from analyses
[c]Pearson's Chi-Square test for categorical variables. Yates' continuity correction was used for 2 by 2 tables.
[d]Performing obstetric ultrasound examinations

For peer review only

For peer review only - http://bmjopen.bmj.com/site/about/guidelines.xhtml

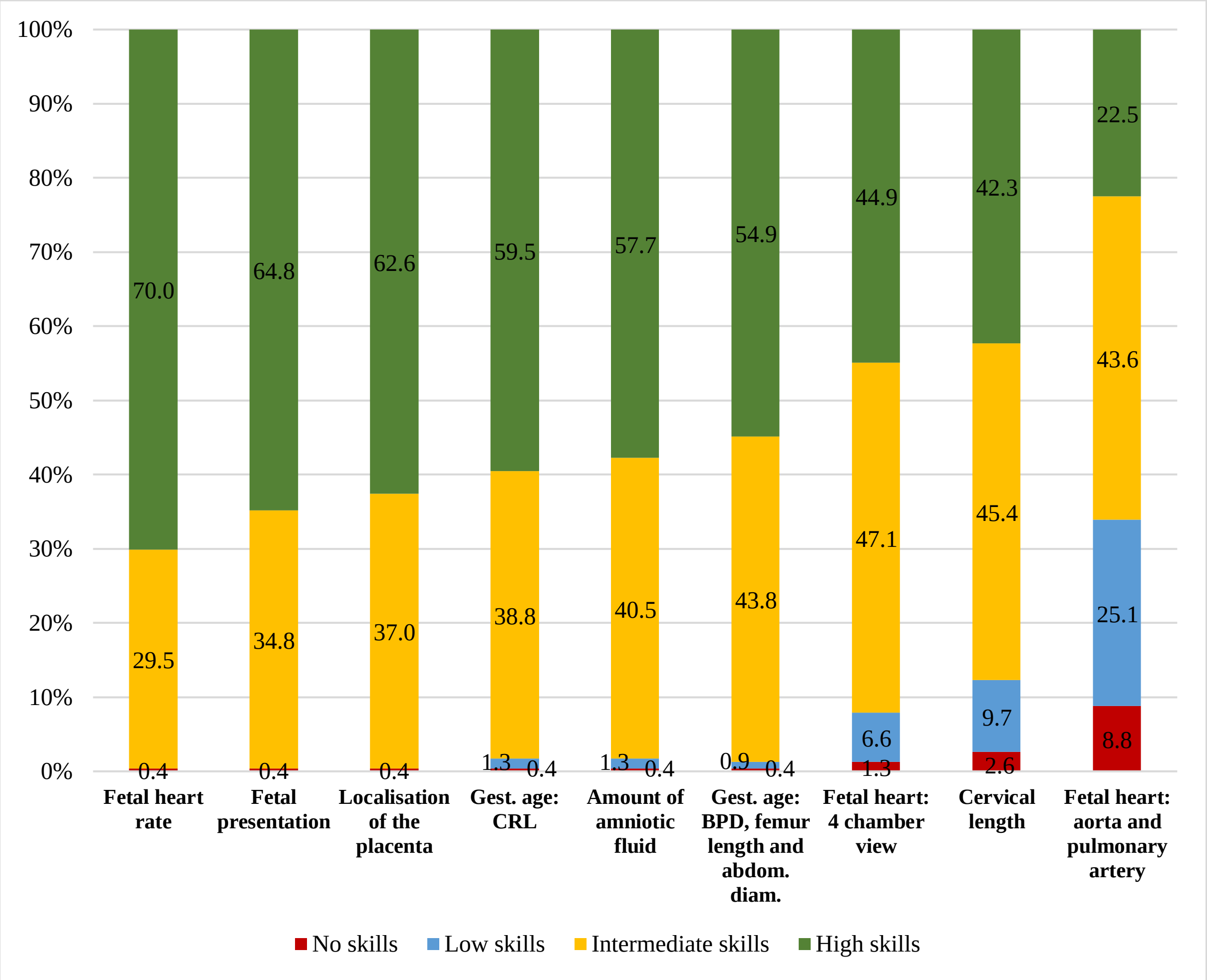
100%
90%
80%
70%
60%
50%
40%
30%
20%
10%
0%
70.0
29.5
0.4
64.8
34.8
0.4
62.6
37.0
0.4
59.5
38.8
1.3
0.4
57.7
40.5
1.3
0.4
54.9
43.8
0.9
0.4
44.9
47.1
6.6
1.3
42.3
45.4
9.7
2.6
22.5
43.6
25.1
8.8
Fetal heart rate
Fetal presentation
Localisation of the placenta
Gest. age: CRL
Amount of amniotic fluid
Gest. age: BPD, femur length and abdom. diam.
Fetal heart: 4 chamber view
Cervical length
Fetal heart: aorta and pulmonary artery
No skills
Low skills
Intermediate skills
High skills

STROBE 2007 (v4) Statement—Checklist of items that should be included in reports of *cross-sectional studies*

Section/Topic	Item #	Recommendation	Reported on page #
**Title and abstract**	1	(*a*) Indicate the study's design with a commonly used term in the title or the abstract	1
		(*b*) Provide in the abstract an informative and balanced summary of what was done and what was found	3-4
**Introduction**			
Background/rationale	2	Explain the scientific background and rationale for the investigation being reported	6-7
Objectives	3	State specific objectives, including any prespecified hypotheses	6-7
**Methods**			
Study design	4	Present key elements of study design early in the paper	8-9
Setting	5	Describe the setting, locations, and relevant dates, including periods of recruitment, exposure, follow-up, and data collection	8-11
Participants	6	(*a*) Give the eligibility criteria, and the sources and methods of selection of participants	10
Variables	7	Clearly define all outcomes, exposures, predictors, potential confounders, and effect modifiers. Give diagnostic criteria, if applicable	11-13
Data sources/ measurement	8*	For each variable of interest, give sources of data and details of methods of assessment (measurement). Describe comparability of assessment methods if there is more than one group	13
Bias	9	Describe any efforts to address potential sources of bias	9-10
Study size	10	Explain how the study size was arrived at	9
Quantitative variables	11	Explain how quantitative variables were handled in the analyses. If applicable, describe which groupings were chosen and why	11-13
Statistical methods	12	(*a*) Describe all statistical methods, including those used to control for confounding	13
		(*b*) Describe any methods used to examine subgroups and interactions	
		(*c*) Explain how missing data were addressed	
		(*d*) If applicable, describe analytical methods taking account of sampling strategy	
		(*e*) Describe any sensitivity analyses	
**Results**			

For peer review only

For peer review only - http://bmjopen.bmj.com/site/about/guidelines.xhtml

Participants	13*	(a) Report numbers of individuals at each stage of study—eg numbers potentially eligible, examined for eligibility, confirmed eligible, included in the study, completing follow-up, and analysed	10, 14
		(b) Give reasons for non-participation at each stage	10
		(c) Consider use of a flow diagram	
Descriptive data	14*	(a) Give characteristics of study participants (eg demographic, clinical, social) and information on exposures and potential confounders	14-15
		(b) Indicate number of participants with missing data for each variable of interest	12, 14-19
Outcome data	15*	Report numbers of outcome events or summary measures	14-20
Main results	16	(*a*) Give unadjusted estimates and, if applicable, confounder-adjusted estimates and their precision (eg, 95% confidence interval). Make clear which confounders were adjusted for and why they were included	13-19
		(*b*) Report category boundaries when continuous variables were categorized	
		(*c*) If relevant, consider translating estimates of relative risk into absolute risk for a meaningful time period	
Other analyses	17	Report other analyses done—eg analyses of subgroups and interactions, and sensitivity analyses	
**Discussion**			
Key results	18	Summarise key results with reference to study objectives	19-20, 24-25
Limitations	19	Discuss limitations of the study, taking into account sources of potential bias or imprecision. Discuss both direction and magnitude of any potential bias	23-24
Interpretation	20	Give a cautious overall interpretation of results considering objectives, limitations, multiplicity of analyses, results from similar studies, and other relevant evidence	24-25
Generalisability	21	Discuss the generalisability (external validity) of the study results	23-24
**Other information**			
Funding	22	Give the source of funding and the role of the funders for the present study and, if applicable, for the original study on which the present article is based	26

*Give information separately for cases and controls in case-control studies and, if applicable, for exposed and unexposed groups in cohort and cross-sectional studies.

Note: An Explanation and Elaboration article discusses each checklist item and gives methodological background and published examples of transparent reporting. The STROBE checklist is best used in conjunction with this article (freely available on the Web sites of PLoS Medicine at http://www.plosmedicine.org/, Annals of Internal Medicine at http://www.annals.org/, and Epidemiology at http://www.epidem.com/). Information on the STROBE Initiative is available at www.strobe-statement.org.

For peer review only

BMJ Open

Health professionals' experiences and views on obstetric ultrasound in Vietnam: a regional, cross-sectional study

Journal:	*BMJ Open*
Manuscript ID	bmjopen-2019-031761.R1
Article Type:	Original research
Date Submitted by the Author:	12-Aug-2019

Complete List of Authors:	Holmlund, Sophia; Umea Universitet Medicinska fakulteten, Clinical Sciences, Obstetrics and Gynecology<br>Pham Thi, Lan; Hanoi Medical University, Department of Dermatology and Venereology<br>Edvardsson, Kristina; Umea Universitet Medicinska fakulteten, Department of Clinical Sciences, Obstetrics and Gynecology; La Trobe University, Judith Lumley Centre, School of Nursing and Midwifery<br>Ho Dang, Phuc ; Vietnam Academy of Science and Technology, Department of Probability and Mathematical Statistics, Institute of Mathematics<br>Ntaganira, Joseph; University of Rwanda College of Medicine and Health Sciences, School of Public Health<br>Small, Rhonda; La Trobe University, Judith Lumley Centre, School of Nursing and Midwifery; Karolinska Institutet, Department of Women's and Children's and Reproductive Health<br>Lesio Kidanto, Hussein; Aga Khan University - Tanzania, Department of Obstetrics and Gynecology<br>Ngarina, Matilda; Muhimbili National Hospital, Department of Obstetrics and Gynecology<br>Mogren, Ingrid; Umeå Universitet Medicinska fakulteten, Department of Clinical Sciences, Obstetrics and Gynecology; La Trobe University, Judith Lumley Centre, School of Nursing and Midwifery

**Primary Subject Heading**:	Obstetrics and gynaecology
Secondary Subject Heading:	Epidemiology, Medical education and training, Public health, Reproductive medicine
Keywords:	prenatal ultrasonography, obstetricians, midwives, prenatal care, pregnancy, Vietnam

SCHOLARONE™
Manuscripts

Health professionals' experiences and views on obstetric ultrasound in Vietnam: a regional, cross-sectional study

Sophia Holmlund[1*], Lan Pham Thi[2], Kristina Edvardsson[1,3], Phuc Ho Dang[4], Joseph Ntaganira[5], Rhonda Small[3,6], Hussein Lesio Kidanto[7], Matilda Ngarina[8], Ingrid Mogren[1,3]

[1]Department of Clinical Sciences, Obstetrics and Gynecology, Umeå University, Umeå, Sweden

[2]Department of Dermatology and Venereology, Hanoi Medical University, Hanoi, Vietnam

[3]Judith Lumley Centre, School of Nursing and Midwifery, La Trobe University, Melbourne, Australia

[4]Department of Probability and Mathematical Statistics, Institute of Mathematics, Vietnam Academy of Science and Technology, Hanoi, Vietnam

[5]School of Public Health, College of Medicine and Health Sciences, University of Rwanda, Kigali, Rwanda

[6]Department of Women's and Children's and Reproductive Health, Karolinska Institutet, Stockholm, Sweden

[7]Department of Obstetrics and Gynecology, Aga Khan University, Dar es Salaam, Tanzania

[8]Department of Obstetrics and Gynecology, Muhimbili National Hospital, Dar es Salaam, Tanzania

Email addresses:

SH: sofia.holmlund@umu.se

LPT: lanphamthi009@gmail.com

KE: k.edvardsson@latrobe.edu.au

PHD: hodang54@yahoo.com

JN: jntaganira@ur.ac.rw

RS: r.small@latrobe.edu.au

HLK: hkidanto@gmail.com

MN: mmatty71@gmail.com

IM: ingrid.mogren@umu.se

***Corresponding author**

For peer review only

Address: Department of Clinical Sciences, Obstetrics and Gynecology, Umeå University, SE 901 87 Umeå, Sweden, sofia.holmlund@umu.se, +46 90-7850463

For peer review only

Abstract

Objectives

Obstetric ultrasound is an important part of antenatal care in Vietnam, although there are great differences in access to antenatal care and ultrasound services across the country. The aim of this study was to explore Vietnamese health professionals' experiences and views of obstetric ultrasound in relation to clinical management, resources and skills.

Design

A cross-sectional questionnaire study was performed as part of the **CRO**ss **C**ountry **U**ltra**S**ound study (CROCUS study).

Setting

Health facilities (N=29) in urban, semi-urban and rural areas of Hanoi region, Vietnam.

Participants

Participants were 289 obstetricians/gynaecologists and 535 midwives.

Results

A majority (88%) of participants agreed that "every woman should undergo ultrasound examination" in pregnancy to determine gestational age. Participants reported an average of six ultrasound examinations as medically indicated during an uncomplicated pregnancy.

For peer review only

Access to ultrasound at participants' workplaces was reported as good regardless of health facility level. Most participants performing ultrasound reported high level skills for fetal heart rate examination (70%), whereas few (23%) reported being skilled in examination of the anatomy of the fetal heart. Insufficient ultrasound training leading to suboptimal pregnancy management was reported by 37% of all participants. "Better quality of ultrasound machines", "more physicians trained in ultrasound" and "more training for health professionals currently performing ultrasound" were reported as ways to improve the utilisation of ultrasound.

Conclusions

Obstetric ultrasound is used as an integral part of antenatal care at all selected health facility levels in the region of Hanoi, and access was reported as high. However, reports of insufficient ultrasound training resulting in suboptimal pregnancy management indicates a need for additional training of ultrasound operators to improve utilisation of ultrasound.

Strengths and limitations of this study

- The study questionnaire was developed based on the results from earlier qualitative studies performed with obstetricians/physicians and midwives/nurses in six different countries (the CROCUS study).
- The strengths of this study include participants of different health professional categories recruited from different levels of the health care system in urban, semi-urban and rural areas of Hanoi.
- The research team comprised two Vietnamese researchers familiar with the setting and the health care system which strengthens the interpretation of data.

For peer review only

- One limitation of this study may be the translation of the questionnaire from English to Vietnamese, however, measures to reduce the risk of losing the intended meaning of questions and statements were implemented.
- Since previous studies within this research domain are lacking, the power calculation was based on assumptions of proportions for one outcome variable in relation to one background variable, and may therefore mean uncertainty of the required study sample.

Keywords

prenatal ultrasonography; health personnel; obstetricians; midwives; prenatal care; pregnancy; Vietnam

For peer review only

Background

Sufficient antenatal care (ANC) services and skilled birth attendance are important factors contributing to safer deliveries, reductions in obstetric complications, and decreased maternal and neonatal morbidity and mortality [1]. Timely and appropriate evidence-based practices in ANC including health promotion, screening and diagnosis, and prevention for diseases can save lives [2]. Since 2016, the World Health Organization (WHO) recommends a minimum of eight ANC contacts during pregnancy and one ultrasound examination before 24 weeks of gestation. The aim of the recommendation of an early ultrasound scan is to estimate gestational age, improve detection of multiple pregnancy and fetal anomaly, reduce induction of post-term pregnancy and improve women's pregnancy experience [2]. Ultrasound is an important part of ANC in high income countries [3], and clinical trials show that ultrasound may improve management and pregnancy outcomes in the developing world [4].

The utilisation of reproductive health services in Vietnam has substantially increased over recent decades, but there are still inequities in the country [5]. Low education, poverty, ethnic minority status [6] and living in rural areas are factors associated with decreased access to reproductive health services [5]. Ultrasound has become a central tool in ANC services in Vietnam [7]. Currently the Ministry of Health in Vietnam recommends at least four ANC visits [8], in accordance with the previous recommendation by WHO [9]. Additionally, three ultrasound examinations are recommended during pregnancy, in gestational week 11-13, 20-24 and 30-32 [8]. In 2014, more than 70% of pregnant women in Vietnam received at least four ANC visits [10]. However, almost half of these women reported not having urine or blood samples taken or blood pressure measurement done during their last pregnancy [10]. The private health sector is a continuously growing part of health care services in Vietnam, especially for provision of legal abortion services and obstetric ultrasound examinations [11]. Limited salary

For peer review only

for physicians creates incentives for both public and private practitioners to search for additional income through provision of these services [12 13]. Commercialisation of ultrasound services has led to urban Vietnamese women having an average of more than six ultrasound scans during pregnancy which is a high number of examinations from an international perspective [12 14].

Study rationale

Few studies have investigated the use of obstetric ultrasound in Vietnam from health professionals' perspectives, although ultrasound is frequently used during pregnancy. This study serves to fill a knowledge gap and can benefit authorities in their work to further develop education and improvement of guidelines on obstetric ultrasound use. This study is part of the **CRO**ss **C**ountry **U**ltra**S**ound study (CROCUS) investigating health professionals' experiences and views of the use of ultrasound in high-, middle and low-resource countries.

Aims

The overall aim of this study was to explore different aspects of obstetric ultrasound in Vietnam from health professionals' perspectives.

The research questions investigated were:

- What are health professionals' views of the role of obstetric ultrasound for clinical management of pregnancy?
- How do health professionals view access to obstetric ultrasound?
- How do health professionals assess their skills in performing obstetric ultrasound examinations?

For peer review only

- What do health professionals believe could improve the utilisation of obstetric ultrasound?

Materials and methods

The Vietnamese setting

Since 2010, Vietnam has been classified as a low to middle income country and has undergone substantial economic development in recent decades [1]. The maternal mortality rate has decreased from 139/100 000 in 1990 to 54/100 000 live births in 2015 [15]. Most inpatient health care is provided by public hospitals but for outpatient care, private clinics account for a large number of patients [16]. Vietnam is divided into 63 provinces, 698 districts and 11,121 communes [17]. In each commune, a village health worker (VHW) provides health promotion, immunisation, nutrition services and attends births in remote areas [18]. At the community health centre level (CHS), a midwife or an assistant doctor is in charge of maternal health services and provides ANC, assists normal delivery, and provides postnatal care, immunisation services and supervision of the VHW. At district level, ANC, delivery care including caesarean sections and newborn care are provided at hospitals [18] while maternity homes deliver basic prenatal and delivery services [16 18]. Provincial hospitals provide more specialised health care, and referrals from lower health care levels to provincial level are undertaken if complications occur during pregnancy or delivery [18]. At the top of the health care system there are several national hospitals providing specialised care and receiving referrals from lower levels [16].

Study design

For peer review only

This cross-sectional study used a questionnaire to investigate a number of research questions related to obstetric ultrasound with obstetricians/gynaecologists and midwives providing pregnancy, delivery and postpartum care to women in the region of Hanoi, Vietnam.

Sampling

Owing to the lack of findings from similar studies, a sample size of 290 obstetricians/gynaecologists and a corresponding number of midwives (n=290) was calculated based on plausible estimations of prevalence of background characteristics and outcome variables. The calculation was based on the outcome requiring the largest sample size "every woman should undergo ultrasound examination in pregnancy to determine gestational age" and the background variable "work experience over and under 5 years", to detect a difference in proportion of 0.10 with a power of 80% and a significance level of 5%.

Purposive sampling was used to obtain a representative sample of health professionals caring for pregnant women at different levels of health facilities in urban, semi-urban and rural areas in the region of Hanoi. One national hospital, one provincial hospital, 24 district hospitals and three maternity homes were included in the study, in total 29 health facilities.

Questionnaire

The study questionnaire was developed based on the results from the earlier qualitative studies performed in the CROCUS study [7 19-27]. Sociodemographic characteristics, evaluation of self-reported skills in performing ultrasound and questions about access to obstetric ultrasound and health professionals' views on what may improve utilisation of ultrasound in Vietnam were included, among other items. The questionnaire included both questions and statements, and the items had either fixed or Likert-scale response options. This analysis investigates the research questions noted above, utilising 45 of the 105 questions and

For peer review only - http://bmjopen.bmj.com/site/about/guidelines.xhtml

statements. The questionnaire was developed first in English and thereafter translated to Vietnamese by a native Vietnamese speaker independent of the research team. The Vietnamese version of the questionnaire was also back-translated to English by another external person. This check resulted in minor adjustments of some words, but demonstrated that the Vietnamese translation had retained the overall meaning of the English. The questionnaire was pilot-tested with 10 obstetricians, 6 midwives and 2 sonographers. No further revisions of the questionnaire were required as a result of piloting.

Data collection procedures including recruitment of study participants

The data collection was performed in April 2017 by four experienced data collectors supervised by two Vietnamese senior researchers in the research team. Before the start of the data collection, data collectors were trained by the research team and all questions and statements in the questionnaire were discussed to ensure correct understanding. The two Vietnamese researchers initiated contact with the directors of all selected health facilities and all of them agreed to assist with recruitment of participants. For this study, we aimed to include health professionals caring for pregnant women and with different experiences in relation to use of obstetric ultrasound. Eligible participants were health professionals managing pregnant women at the maternity wards on the day of data collection at each study site. No eligible participant declined participation in the study. The primary sample included 890 participants. Six individuals working as radiology technicians were excluded from the primary sample as the they did not fulfil the inclusion criteria, and 60 sonographers were also excluded from the primary sample as suggested by the journal. The final sample (N=824) included the following health professionals: obstetricians/gynaecologists (n=289) and midwives (n=535). Participation was anonymous and all questionnaires were given a unique

For peer review only

code. Safe storage of questionnaires was undertaken in accordance with national procedures and regulations. Data were entered into an SPSS file at the Hanoi Medical University, by two experienced data clerks. To evaluate the quality of the data entry, every 10th questionnaire based on the number order of identification codes, was selected for data re-entry. The data from all 107 variables in 89 questionnaires were re-entered in the SPSS file by the first author. The rate of error was 1.4 %. The identified errors in the SPSS file were corrected.

Patient and Public Involvement

This research was done without patient involvement.

Independent variables

Age was calculated as a continuous variable using birth year and year of data collection. For some analyses age was dichotomised as age 34 years or less and 35 years and above. *Gender* included female or male. *Health profession* included the following response options in the questionnaire: obstetrician/gynaecologist, general practitioner, resident physician, physician other (please specify), midwife, radiologist/sonographer and “other” (please specify). *Health profession* was thereafter categorised into two groups: obstetricians/gynaecologists and midwives. Resident physicians undergoing post-graduate training (n=9) and general practitioners (n=12) were also included in the category obstetricians/gynaecologists because they worked at the same department and performed similar work tasks as the obstetricians/gynaecologists. One participant who was an anaesthesiologist by profession, but was working with maternity care, was categorised as an obstetrician/gynaecologist. One nurse working in maternity care was categorised as a midwife. *Health facilities* included the response options national hospital, provincial hospital, district hospital and maternity home. The variable *health facilities* was dichotomised into national hospital/provincial hospital and

For peer review only

district hospital/maternity home in some analyses. *Area of health facility* was categorised as hospitals in urban (n=7), semi-urban (n=5) and rural (n=17) areas of Hanoi. *Type of health care* was classified as public, private and both public and private health care. No participant reported working only in private health care. *Number of ultrasound examinations indicated in an uncomplicated pregnancy* was categorised based on the three recommended number of ultrasound examinations by the Ministry of Health in Vietnam; 3 examinations or less and 4 examinations or more.

For peer review only

Dependent variables

The dependent variables with fixed response alternatives are presented in Table 1. For the statements related to “the role of ultrasound for clinical management” and “resources and training of obstetric ultrasound”, the response options were dichotomised into *disagree* or *strongly disagree* and *agree* or *strongly agree* in logistic regression analyses. The question “do you have a role in decision-making regarding clinical management on the basis of obstetric ultrasound examinations” was both used as an independent and dependent variable and the response options were dichotomised into *no* and *yes* for some analyses. The response options of the statements related to “improving utilisation of ultrasound” were categorised as *not at all* or *not very much* and *a fair amount* or *a great deal* in logistic regression analyses. The response option *neutral* or *don´t know* was not included in either of these categories. For most statements, the response options *neutral* and *don´t know* were selected by a small proportion of the participants.

Table 1. Questions or statements and their response options in the questionnaire

How often do you perform obstetric ultrasound examinations? [a]
How do you rate your skills in ultrasound in relation to the assessment/evaluation of:<br>• Fetal presentation[b]<br>• Localisation of the placenta[b]<br>• Fetal heart rate[b]<br>• Amniotic fluid amount[b]

• Gestational age estimated by CRL (crown-rump-length)[b]<br>• Gestational age estimated by biparietal diameter, femur length and abdominal diameter[b]<br>• Cervical length[b]<br>• Fetal heart: 4 chamber view[b]<br>• Fetal heart: aorta and pulmonary artery[b]<br>• Doppler: umbilical artery[b]
Do you have a role in decision-making regarding clinical management on the basis of obstetric ultrasound examinations?[c]
How often do you make decisions based on the results from obstetric ultrasound examinations in your clinical work? [a]
What do you believe would improve the utilisation of ultrasound at your clinic/work place?<br>• More ultrasound machines[d]<br>• Better quality of ultrasound machines[d]<br>• More training for health professionals currently performing ultrasound[d]<br>• More doctors trained in ultrasound[d]<br>• (More) midwives trained in ultrasound[d]
Statements on ultrasound resources and training<br>• Pregnant women in my country have access to dating ultrasound (i.e. estimation of gestational age)[e]<br>• Pregnant women in my country have access to fetal anomaly screening[e]<br>• Pregnant women in my country have access to obstetric ultrasound independent of area of living[e]<br>• Pregnant women in my country have access to obsteric ultrasound independent of income[e]<br>• There are enough resources in my country to provide *medically indicated* obstetric ultrasound examinations to pregnant women who need it[e]<br>• At my workplace, there is always access to obstetric ultrasound when it is needed[e]<br>• At my workplace, lack of ultrasound training of the ultrasound operator sometimes leads to suboptimal pregnancy management[e]<br>• Maternity care in my country would improve if midwives were qualified to perform basic ultrasound examinations[e]
Statements on the role of ultrasound in clinical management of pregnancy<br>• Ultrasound is decisive in pregnancy management[e]<br>• Every woman should undergo ultrasound examination in pregnancy to determine gestational age[e]<br>• It is irresponsible of a pregnant woman to decline a dating scan[e]<br>• Ultrasound is safe to use for the pregnant woman and the fetus irrespective of the number of examinations[e]<br>• Ultrasound is important for expectant parents to bond with their fetus during pregnancy[e]

[a]Response options: Never, On a daily basis, On a weekly basis, On a monthly basis, More seldom than on a monthly basis
[b]Response options: No skills, Skill-level low, Skill-level intermediate, Skill-level high
[c]Response options: No, Yes a minor role, Yes a moderate role, Yes a major role
[d]Response options: Not at all, Not very much, A fair amount, A great deal, Don't know
[e]Response options: Strongly agree, Agree, Neutral, Disagree, Strongly disagree

Statistical analysis

For categorical variables, frequencies and percentages were analysed and Pearson's Chi-Square test was used for test of difference, with the level of significance set at $p<0.05$. For continuous variables, mean values and their standard deviations (SD) were presented. Univariable and multivariable logistic regression was undertaken and are presented with odds ratios (OR) and their 95% confidence intervals (CI). The independent and dependent variables used for logistic regression are reported in their specific sections above as well as in Table 1. All independent variables were entered into the univariable logistic regression analysis,

For peer review only

however only the statistically significant variables were included in the final multivariable logistic regression models. Statistical analyses were performed using SPSS version 23.

Results

Background characteristics of the study sample

A total of 824 participants aged 21-60 years (mean age 34.8 years) were enrolled in the study. The distribution of health professionals was obstetricians/gynaecologists (35.1%), and midwives (64.9%; Table 2). One third of the participants (28.1%) was performing obstetric ultrasound and mainly on a daily basis (66.5%). All obstetricians/gynaecologists working in maternity homes (100%) were performing ultrasound as well as a majority of obstetricians/gynaecologists working in provincial hospitals (84.9%), district hospitals (75.2%) and national hospitals (75.7%). The mean estimated number of ultrasound examinations per day was 15.7 (median 10, range 1-100) for obstetricians/gynaecologists. Obstetricians/gynaecologists performing more than ten examinations per day were significantly older (≥35 years) than those obstetricians/gynaecologists performing ten or fewer examinations per day ($p<0.001$). A few participants at all health care levels (8.3%) reported that midwives performed ultrasound in their workplace. Participants at district hospitals (7.0%) and maternity homes (6.3%) reported the lowest percentage of midwives performing ultrasound in their workplace.

For peer review only

Table 2. Background characteristics of the study sample (N=824)

Variable	All health professionals<br>Total= 824<br>n (%)	Obstetricians/Gynaecologists<br>Total=289<br>n (%)	Midwives<br>Total=535<br>n (%)
**Age (years)**	***811 (98.4)***	***286 (99.0)***	***525 (98.1)***
Mean; SD[a]	34.8; 8.7	36.6; 9.2	33.7; 8.3
Min-Max	21-60	23-60	21-55
**Years in profession**	***818 (99.3)***	***288 (99.7)***	***530 (99.1)***
Mean; SD[a]	10.5; 8.3	10.4; 8.9	10.5; 8.1
Min-max	0-35	0-32	0.5-35
**Years in health care**	***817 (99.2)***	***287 (99.3)***	***530 (99.1)***
Mean; SD[a]	11.1; 8.5	11.6; 9.2	10.9; 8.2
Min-max	0-38	0-38	0.5-35
**Gender**	***824 (100)***	***289 (100)***	***535 (100)***
Male	123 (14.9)	123 (42.6)	0
Female	701 (85.1)	166 (57.4)	535 (100.0)
**Marital status**	***817 (99.2)***	***287 (99.3)***	***530 (99.1)***
Married	714 (87.4)	242 (84.3)	472 (89.1)
Separated/Divorced	1 (0.1)	0 (0.0)	1 (0.2)
Widowed	4 (0.5)	2 (0.7)	2 (0.4)
Not married/Single	98 (12.0)	43 (15.0)	55 (10.4)
**Having children**	***821 (99.6)***	***288 (99.7)***	***533 (99.6)***
Yes	684 (83.3)	230 (79.9)	454 (85.2)
No	137 (16.7)	58 (20.1)	79 (14.8)
**Type of health care**	***823 (99.9)***	***289 (100)***	***534 (99.8)***
Public	789 (95.9)	268 (92.7)	521 (97.6)
Both public and private	34 (4.1)	21 (7.3)	13 (2.4)
**Level of health facility[b]**	***824 (100)***	***289 (100)***	***535 (100)***
National hospital	144 (17.5)	74 (25.6)	70 (13.1)
Provincial hospital	184 (22.3)	86 (29.8)	98 (18.3)
District hospital	464 (56.3)	121 (41.9)	343 (64.1)
Maternity home	32 (3.9)	8 (2.8)	24 (4.5)
**Area of health facility[c]**	***824 (100)***	***289 (100)***	***535 (100)***
Urban	439 (53.3)	191 (66.1)	248 (46.4)
Semi-urban	129 (15.7)	35 (12.1)	94 (17.6)
Rural	256 (31.1)	63 (21.8)	193 (36.1)
**Provision of maternity services[d]**			
Antenatal care	683 (83.0)	261 (90.3)	422 (79.0)
Intrapartum care	642 (78.0)	245 (84.8)	397 (74.3)
Postpartum care	688 (83.6)	235 (81.3)	453 (84.8)
Do not currently provide maternity care	32 (3.9)	10 (3.5)	23 (4.3)
**Performing ultrasound[e]**	***823 (99.9)***	***289 (100)***	***534 (99.8)***
Yes	231 (28.1)	228[f] (78.9)	3 (0.6)
No	592 (71.9)	61 (21.1)	531 (99.4)

[a]SD= Standard Deviation
[b]Number of participants at specified health facilities
[c]Number of participants at specified areas of health facilities
[d]Item in questionnaire: "Which of the following maternity services do you provide? (Please tick all that apply)"
[e]Performing obstetric ultrasound examinations
[f]One participant have not rated the skills in relation to different tasks during ultrasound examinations

For peer review only

The role of obstetric ultrasound

Most participants (66.2%) agreed or strongly agreed that "ultrasound is decisive in pregnancy management". Obstetricians/gynaecologists reported significantly lower agreement (55.7%) with the statement "ultrasound is decisive in pregnancy management" than midwives (71.8%; $p<0.001$). A majority (87.5%) of participants, independent of health profession, agreed or strongly agreed that "every woman should undergo ultrasound examination to determine gestational age". Most participants (75.0%) agreed or strongly agreed that "it is irresponsible of a pregnant woman to decline a dating ultrasound" and there was no significant difference in opinion between different health professionals. There was a significant difference in opinion between the different health professionals for the statement "ultrasound is safe to use for the woman and the fetus irrespective of the number of examinations", where obstetricians/gynaecologists were more likely to agree or strongly agree than midwives (OR 1.96; 95% CI 1.22-3.17). Participants reported an average of 5.9 ultrasound examinations as medically indicated during an uncomplicated pregnancy (obstetricians/gynaecologists: SD 2.7, range 2-15, and midwives: SD 2.6, range 2-20). A quarter of the ultrasound operators (25.1%) agreed with the national guidelines that three ultrasound examinations are medically indicated during an uncomplicated pregnancy. Midwives in comparison to obstetricians/gynaecologists, were more likely to agree or strongly agree that "ultrasound is important for expectant parents to bond with their fetus during pregnancy" (OR 1.59; 95% CI 1.13-2.26). The assessment that four ultrasound examinations or more are medically indicated in an uncomplicated pregnancy was associated with higher agreement with the statement "ultrasound is important for expectant parents to bond with their fetus during pregnancy", compared to those assessing that three ultrasound examinations or fewer are medically

For peer review only

indicated in a normal pregnancy (OR 1.61; 95% CI 1.03-2.50, adjusted for health profession and performing ultrasound or not).

Access to obstetric ultrasound

Most of the participants (95.6%-100%), regardless of health facility level, agreed or strongly agreed with the statement "there is always access to obstetric ultrasound when needed at my workplace". Almost all participants (95.4%-100%) at all health facility levels reported that they agreed or strongly agreed with the statement "pregnant women in the country have access to dating ultrasound". A majority of participants at all health facility levels (93.8%-95.1%), agreed or strongly agreed that "pregnant women in the country have access to fetal anomaly screening". Midwives were more likely to agree or strongly agree that "pregnant women in the country have access to ultrasound independent of area of residence, compared with obstetricians/gynaecologists (OR 2.54; 95% CI 1.60-4.02). Participants in national hospitals and provincial hospitals reported significantly lower agreement with the statement "pregnant women in my country have access to ultrasound independent of income" than participants in district hospitals and maternity homes ($p<0.001$). Further results are presented in Table 3.

Table 3. Health professionals' views on specified statements[a] (N=824)

Statement	Obstetricians/ Gynaecologists Total=289[c] n (%)	Midwives Total=535[c] n (%)	p-value[b]
**Pregnant women in my country have access to dating ultrasound (i.e. estimation of gestational age)**	***279 (96.5)***	***521 (97.4)***	0.98
Agree/Strongly agree	275 (98.6)	512 (98.3)	
Disagree/Strongly disagree	4 (1.4)	9 (1.7)	
**Pregnant women in my country have access to fetal anomaly screening**	***276 (95.5)***	***514 (96.1)***	0.16
Agree/Strongly agree	269 (97.5)	509 (99.0)	
Disagree/Strongly disagree	7 (2.5)	5 (1.0)	
**Pregnant women in my country have access to obstetric ultrasound independent of area of residence**	***248 (85.8)***	***485 (90.7)***	<0.001

For peer review only

Agree/Strongly agree	203 (81.9)	446 (92.0)	
Disagree/Strongly disagree	45 (18.1)	39 (8.0)	
**Pregnant women in my country have access to obstetric ultrasound independent of income**	***228 (78.9)***	***475 (88.8)***	0.082
Agree/Strongly agree	178 (78.1)	398 (83.8)	
Disagree/Strongly disagree	50 (21.9)	77 (16.2)	
**There are enough resources in my country to provide medically indicated obstetric ultrasound examinations to pregnant women who need it**	***234 (81.0)***	***447 (83.6)***	0.002
Agree/Strongly agree	185 (79.1)	395 (88.4)	
Disagree/Strongly disagree	49 (20.9)	52 (11.6)	
**At my workplace, there is always access to obstetric ultrasound when it is needed**	***282 (97.6)***	***519 (97.0)***	0.010
Agree/Strongly agree	272 (96.5)	515 (99.2)	
Disagree/Strongly disagree	10 (3.5)	4 (0.8)	
**At my workplace, lack of ultrasound training of the ultrasound operator sometimes leads to suboptimal pregnancy management**	***237 (82.0)***	***452 (84.5)***	0.086
Agree/Strongly agree	115 (48.5)	187 (41.4)	
Disagree/Strongly disagree	122 (51.5)	265 (58.6)	

[a]The response option *neutral* was excluded from analyses
[b]Pearsons Chi-Square Test with Yates' continuity correction for test of differences in proportion between the two groups of obstetricians/gynaecologists and midwives
[c]The number of participants included in each analysis are presented in relation to the total sample of each group of obstetricians/gynaecologists and midwives

Ultrasound operators' decision-making and self-rated skills

Almost all obstetricians/gynaecologists (92.2%) and a majority of midwives (59.4%) reported having a role in decision-making regarding clinical management on the basis of obstetric ultrasound examinations. There was a significant difference in the proportion of obstetricians/gynaecologists (38.6%) and midwives (20.5%) reporting having a major role in decision-making compared to having a minor or moderate role in decision-making (p<0.001). Approximately one third of all health professionals (37.1%) reported that they agreed or strongly agreed with the statement "at my workplace, lack of ultrasound training of the ultrasound operator sometimes leads to suboptimal pregnancy management". Health professionals performing ultrasound were asked to rate their skills in relation to different tasks during ultrasound examinations (Figure 1). For all the obstetricians/gynaecologists performing ultrasound (n=228), fetal heart rate was the examination where most participants

For peer review only

reported high skills (70.0%) and examination of the fetal heart: aorta and pulmonary artery was the examination with the lowest proportion of self-rated high skill level (22.5%).

Figure 1.

Improving utilisation of obstetric ultrasound

When health professionals were asked to assess how much particular strategies could improve the utilisation of ultrasound, “better quality of ultrasound machines” (94.0%), “more training for health professionals currently performing ultrasound” (92.8%) and “more physicians trained in ultrasound” (93.2%) were the statements with the highest numbers of participants reporting a fair amount or a great deal. Obstetricians/gynaecologists were more likely to agree that “more training for health professionals currently performing ultrasound would improve the utilisation of ultrasound” compared to midwives (OR 2.60; 95% CI 1.24-5.46). Further results are presented in Table 4. A majority of participants (69.0%) agreed or strongly agreed with the statement “maternity care in my country would improve if midwives were qualified to perform basic ultrasound examinations”, however midwives were more likely to agree or strongly agree than obstetricians/gynaecologists (OR 5.09; 95% CI 3.41-7.61). Participants working in rural hospitals (79.6%) and semi-urban hospitals (78.3%) were also more likely to agree with the previous statement than participants working in urban hospitals (60.0%) ($p<0.001$).

For peer review only

Table 4. Health professionals' views on factors that may improve utilisation of obstetric ultrasound, presented through preformed statements

Variable	More ultrasound machines[a]			Better quality of ultrasound machines[a]		
	Not at all or Not very much[b] **n (%)**	A fair amount or A great deal[b] **n (%)**	p-value[c]	Not at all or Not very much[b] **n (%)**	A fair amount or A great deal[b] **n (%)**	p-value[c]
**Health profession**	***149 (20.1)***	***593 (79.9)***	0.042	***40 (5.2)***	***732 (94.8)***	0.036
Obstetricians/Gynaecologists	65 (24.3)	203 (75.7)		8 (2.8)	276 (97.2)	
Midwives	84 (17.7)	390 (82.3)		32 (6.6)	456 (93.4)	
**Level of health facility**	***149 (20.1)***	***593 (79.9)***	0.003	***40 (5.2)***	***732 (94.8)***	0.232
National hospital	31 (24.4)	96 (75.6)		3 (2.2)	135 (97.8)	
Provincial hospital	43 (28.3)	109 (71.7)		12 (7.2)	154 (92.8)	
District hospital	73 (16.9)	359 (83.1)		24 (5.5)	413 (94.5)	
Maternity home	2 (6.5)	29 (93.5)		1 (3.2)	30 (96.8)	
**Performing ultrasound[d]**	***149 (20.1)***	***592 (79.9)***	0.045	***40 (5.2)***	***731 (94.8)***	0.060
Yes	53 (25.0)	159 (75.0)		6 (2.6)	221 (97.4)	
No	96 (18.1)	433 (81.9)		34 (6.3)	510 (93.8)	
	**More training for health professionals currently performing ultrasound[a]**			**More physicians trained in ultrasound[a]**		
	Not at all or Not very much[b] **n (%)**	A fair amount or A great deal[b] **n (%)**	p-value[c]	Not at all or Not very much[b] **n (%)**	A fair amount or A great deal[b] **n (%)**	p-value[c]
**Health profession**	***48 (6.3)***	***717 (93.7)***	0.014	***43 (5.7)***	***714 (94.3)***	1.000
Obstetricians/Gynaecologists	9 (3.2)	269 (96.8)		16 (5.7)	263 (94.3)	
Midwives	39 (8.0)	448 (92.0)		27 (5.6)	451 (94.4)	
**Level of health facility**	***48 (6.3)***	***717 (93.7)***	0.097	***43 (5.7)***	***714 (94.3)***	0.047
National hospital	6 (4.5)	126 (95.5)		3 (2.3)	129 (97.7)	
Provincial hospital	15 (9.1)	149 (90.9)		15 (9.2)	148 (90.8)	
District hospital	23 (5.2)	416 (94.8)		22 (5.1)	410 (94.9)	
Maternity home	4 (13.3)	26 (86.7)		3 (10.0)	27 (90.0)	
**Performing ultrasound[d]**	***48 (6.3)***	***716 (93.7)***	0.034	***43 (5.7)***	***713 (94.3)***	0.950
Yes	7 (3.2)	215 (96.8)		12 (5.4)	211 (94.6)	
No	41 (7.6)	501 (92.4)		31 (5.8)	502 (94.2)	
	**(More) midwives trained in ultrasound[a]**					
	Not at all or Not very much[b]	A fair amount or A great deal[b]	p-value[c]			

For peer review only

	n (%)	n (%)	
**Health profession**	***277 (37.5)***	***462 (62.5)***	<0.001
Obstetricians/Gynaecologists	165 (60.7)	107 (39.3)	
Midwives	112 (24.0)	355 (76.0)	
**Level of health facility**	***277 (37.5)***	***462 (62.5)***	0.003
National hospital	50 (38.2)	81 (61.8)	
Provincial hospital	79 (49.4)	81 (50.6)	
District hospital	139 (33.4)	277 (66.6)	
Maternity home	9 (28.1)	23 (71.9)	
**Performing ultrasound**[d]	***277 (37.5)***	***461 (62.5)***	<0.001
Yes	135 (62.2)	82 (37.8)	
No	142 (27.3)	379 (72.7)	

[a]Item in questionnaire: "What do you believe would improve the utilisation of ultrasound at your clinic/work place?"
[b]The response option *don´t know* was excluded from analyses
[c]Pearson's Chi-Square test for categorical variables. Yates' continuity correction was used for 2 by 2 tables.
[d]Performing obstetric ultrasound examinations

For peer review only

Discussion

The main findings of this study are that access to obstetric ultrasound was generally reported as satisfactory regardless of participants' health profession and health facility level. Participants reported an average of almost six ultrasound examinations as medically indicated in an uncomplicated pregnancy in contrast to the three ultrasound examinations that are recommended in the Vietnamese national guidelines. Obstetricians/gynaecologists reported high self-rated skill levels for most obstetric ultrasound examinations, although one third of all health professionals reported that "lack of ultrasound training sometimes leads to suboptimal pregnancy management". "Better quality of ultrasound machines", "more training for health professionals' currently performing ultrasound" and "more physicians trained in ultrasound" were reported as factors that could improve the utilisation of ultrasound to the greatest extent. For participants at health facilities in rural and semi-urban areas compared to those in urban areas, the great majority reported that "maternity care in my country would improve if midwives were qualified to perform basic ultrasound examinations".

The role of obstetric ultrasound

Obstetric ultrasound is one of the most important technological advances in pregnancy surveillance [28]. The majority of health professionals in our study agreed or strongly agreed with the statement "ultrasound is decisive in pregnancy management" but unexpectedly one third of our participants were neutral or disagreed with this statement. Obstetricians/gynaecologists who mainly performed ultrasound reported lower agreement with the above statement than midwives who mainly did not perform ultrasound examinations. CROCUS-study findings in Rwanda also demonstrated that health professionals not themselves performing ultrasounds have more liberal attitudes towards ultrasound use then health professionals performing these examinations [29]. Health

For peer review only

professionals sometimes fear that "routinisation" of ultrasound as an unquestioned and integral part of pregnancy management can exert a negative impact on pregnant women's informed consent, and that its increasing use for fetal examination may have consequences for how disability is viewed in society [25 26 30]. The use of ultrasound may also reduce attention on important clinical parameters such as measurement of blood pressure and proteinuria [24 31]. Previous research from Vietnam has shown that many ultrasound examinations are performed just for reassurance of fetal wellbeing [12]. Although problems of access to obstetric ultrasound exist in many low-resource countries, inappropriate use of ultrasound examinations with no effect on pregnancy outcomes still occurs. In a study from Uganda, more than half of all ultrasound examinations performed were classified as inappropriate, for example dating of pregnancy in a suboptimal gestational week or requesting an ultrasound without medical indication [32]. The Ministry of Health in Vietnam recommends three ultrasound examinations including one examination also in the third trimester. However, a routine late pregnancy ultrasound in unselected or low-risk populations has been reported to have no benefit for mother or baby [33]. It is known that Vietnamese women generally undergo many ultrasound examinations during pregnancy [12]. Although it is well known that ultrasound examinations hold a strong appeal for pregnant women [34], the high number of examinations that pregnant women receive seems also to be supported by the ultrasound operators, as the participants in this study considered that twice as many ultrasound examinations were medically indicated compared to the recommendations by the Ministry of Health, Vietnam [8]. Further, participants' report of a mean number of almost six ultrasound examinations to be medically indicated is also in great contrast to the Cochrane review supporting the 2016 WHO ANC guideline recommending a single ultrasound examination before 24 weeks of gestation [2 35]. Health professionals performing ultrasound in our study also reported performing up to 100 scans per day per ultrasound operator. High workload can lead to inadequate provision of information

For peer review only

to women by the physician, including about indication for ultrasound and the results of the ultrasound examination [12 36]. In a broader perspective, non-medical ultrasound examinations during pregnancy consume resources unnecessarily, with negative impact on other maternal health care services. Issuing medical guidelines stating clear indications for ultrasound surveillance during pregnancy are therefore important and may contribute to more appropriate allocation of resources within the health care system in Vietnam.

Access to obstetric ultrasound

Health professionals in our study generally reported satisfactory access to obstetric ultrasound when needed in their own workplace. Access to ultrasound has increased significantly in many resource-limited settings although there are still large differences in access within and between countries [37]. Our study was performed in the area around Hanoi, i.e. the capital of Vietnam, and it is plausible to believe that access to obstetric ultrasound is higher in this region than in other parts of Vietnam. In Hanoi, ultrasound scans are easily accessible and affordable for most pregnant women both within the public and private health care systems. Increased ANC attendance and health facility delivery rates have been seen in Vietnam in recent years, but also increased inequities in maternity care utilisation [38 39], primarily among women with multiple socioeconomic vulnerabilities [38].

Ultrasound operators' skills

Participants performing ultrasound in our study reported high or intermediate skill levels for the majority of the specified ultrasound examinations. Proper training of health professionals performing ultrasound is critically important [28] especially since ultrasound is operator dependent to a large extent [4]. Further, ultrasound training should include ethics, discuss use and misuse of the ultrasound tool in addition to the quality of ultrasound performance and

For peer review only

clinical implications of its use [28], to ensure maximum diagnostic utility and high levels of sensitivity and specificity [4].

In our study, one third of all health professionals believed that insufficient training of the ultrasound operator sometimes leads to suboptimal pregnancy management. Another study from Vietnam reports that physicians experience less knowledge about fetal anomalies, lack advanced training and do not have appropriate equipment and professional protocols that could support their practice in performing obstetric ultrasound [40]. Inadequately skilled health professionals may cause inadvertent harm, for example, by providing false-positive diagnosis where termination of pregnancy may be an option. Alternatively, false-negative information because of inadequate ability to recognise the signs of important diagnoses can result in parents not being offered further investigations and being inappropriately reassured that everything is normal [41].

Improving utilisation of ultrasound

Our results indicate an increased number of physicians to become appropriately educated in ultrasound examinations, regular in-service training sessions and better quality of ultrasound equipment. Lack of training of health care providers has been seen as the most common barrier to regular ultrasound use, although lack of equipment and maintenance and costs for machines also are explanatory factors [42]. A review of ultrasound training in low and middle income countries (LMICs) shows that health professionals often do not meet the WHO criteria in relation to number of scans, supervision and length of training [43]. The obstacles to ultrasound training in LMICs include lack of time for training because of limited possibilities for absence from the workplace, and the logistics to access qualified teachers [43]. Our results also indicate that health professionals in Vietnam seem to have a very substantial workload. It

For peer review only

has been shown that training of midwives in basic obstetric ultrasound may significantly improve pregnancy management [44-46]. In our study, the majority of midwives were positive to the idea of training midwives in ultrasound to improve utilisation of ultrasound. For rural and semi-urban hospitals, a majority of all participants were also positive to ultrasound-trained midwives. However, obstetricians/gynaecologists did not support the idea of training midwives in ultrasound as much as participating midwives. As in much of Vietnamese society, health professions are organised hierarchically. Nurses' roles can be seen as primarily carrying out doctors' orders, not to take their own initiatives [47 48], and it may be a plausible explanation for why physicians defend performing ultrasound as their duty. Shortages of nurses and midwives and lack of quality of training [49 50], may also be additional explanations for physicians not supporting the idea of training midwives in obstetric ultrasound.

Strengths and limitations

The strengths of this study include participants recruited from different levels of the health care system in urban, semi-urban and rural areas of Hanoi. We believe that the wide range of health facilities is likely to be representative for the Vietnamese health care system. The research team comprised two Vietnamese researchers familiar with the setting and the health care system which strengthens the interpretation of data. An additional strength was that four experienced Vietnamese data collectors, familiar with the setting, collected all data. One limitation of this study may be the translation of the questionnaire from English to Vietnamese, with the risk of losing the intended meaning of questions and statements. However, measures to reduce this risk were implemented, including back-translation of the questionnaire. Another limitation might be the unequal distribution between the categories of physicians and midwives in this sample. Although we aimed for equal numbers of each category of health professional, as assessed in the power calculation, all eligible physicians

For peer review only

and midwives working the day of data collection were included in the study. This resulted in a higher number of midwives. Since there is a lack of previous studies within this research domain, the power calculation was based on assumptions of proportions for one outcome variable in relation to one background variable. The assumptions in the power calculations therefore mean uncertainty of the required study sample. However, the results indicate that the sample size was probably adequate for investigating our questions of interest. There was no question in the questionnaire about whether the participant had received any formal ultrasound training and this may be considered a limitation. In Vietnam, it is widely recognised that participants in studies receive payment. All participants were therefore paid with a small sum for answering the questionnaire which may theoretically have affected the willingness of participation in the study.

Conclusions

Obstetric ultrasound is used as an integral part of antenatal care at all selected health facility levels in the region of Hanoi and access to obstetric ultrasound was also reported to be good at all levels. Overall, participants performing obstetric ultrasound reported satisfactory self-rated skill levels. However, report of insufficient ultrasound training resulting in suboptimal pregnancy management in combination with the suggestion for more training to improve utilisation of ultrasound, indicates a need for additional in-service training of ultrasound operators. The proposal of educating midwives in obstetric ultrasound needs to be further evaluated.

Acknowledgements

Our sincere thanks to the participating health professionals for sharing their time and experiences and for the support by the heads of the Departments of Obstetrics and

For peer review only

Gynaecology at the selected health facilities. Thanks go to the data collectors Do Nam Khanh, Nguyen Huyen Tram, Le Vu Thuy Huong and Nguyen Thi Hue for excellent performance of the data collection. We also thank Annika Åhman, Uppsala University, Sweden, for valuable contributions to the questionnaire and Gabriel Granåsen, Umeå University, Sweden for statistical support. We acknowledge the support received from Hanoi Medical University, Hanoi, Vietnam and Umeå University, Umeå, Sweden.

For peer review only

Abbreviations

CROCUS: Cross Country Ultrasound study; ANC: Antenatal care; WHO: World Health Organization; VHW: Village health worker; CHS: Community health centre level; SD: Standard deviation; OR: Odds ratio; CI: Confidence intervals; LMICs: Low and middle income countries.

Footnotes

Authors' contributions

IM is principal investigator of the CROCUS study. SH, LPT, KE, JN, RS, HLK, MN and IM designed the study. SH, KE and IM were the main responsible for developing the questionnaire with input from all co-authors. LPT, PHD, SH, KE and IM organised and performed the data collection together with data collectors. SH conducted the analyses in collaboration with IM, LPT and PHD. SH drafted the primary manuscript in collaboration with IM. All authors have contributed to and approved the final manuscript, tables and figures.

Funding

This research was made possible by grants from Umeå University, Västerbotten County Council, The Swedish Research Council, Sweden (2014-2672), the Swedish Research Council for Health, Working Life and Welfare (Forte) and the European Commission under a COFAS Marie Curie Fellowship (2013-2699).

Competing interests

The authors declare that they have no competing interests.

Ethics approval and consent to participate

Ethical approval was obtained from the Hanoi Medical University Review Board in Bio-Medical Research, reference 141/HMU IRB. The participants received both written and verbal information about the study including that participation was voluntary. All participants signed a consent form before completing the questionnaire.

Data sharing statement

The datasets used and/or analysed during the current study are available from the corresponding author on reasonable request.

For peer review only

References

1. Ministry of Health Viet Nam, Partnership for Maternal, Newborn & Child Health, WHO, World Bank and Alliance for Health Policy and Systems Research. Sucess factors for women´s and children´s health: Viet Nam. Geneva: World Health Organisation, 2014.
2. World Health Organization. WHO recommendations on antenatal care for a positive pregnancy experience. Geneva, 2016.
3. Salomon LJ, Alfirevic Z, Berghella V, et al. on behalf of the ISUOG Clinical Standards Committe. Practice guidelines for performence of the routine mid-trimester fetal ultrasound scan. *Ultrasound Obstet Gynecol (2010)* doi: 10.1002/uog.8831
4. Stanton K, Mwanri L. Global Maternal and Child Health Outcomes: The Role of Obstetric Ultrasound in Low Resource Settings. *World Journal of Preventive Medicine* 2013;1(3):22-29.
5. Duc NHC, Nakamura K, Kizuki M, et al. Trends in inequalities in utilization of reproductive health services from 2000 to 2011 in Vietnam. *Journal of Rural Medicine : JRM* 2015;10(2):65-78. doi: 10.2185/jrm.2902
6. Goland E, Hoa DT, Malqvist M. Inequity in maternal health care utilization in Vietnam. *International journal for equity in health* 2012;11:24. doi: 10.1186/1475-9276-11-24
7. Edvardsson K, Graner S, Thi LP, et al. 'Women think pregnancy management means obstetric ultrasound': Vietnamese obstetricians' views on the use of ultrasound during pregnancy. *Glob Health Action* 2015;8(1):28405. doi: 10.3402/gha.v8.28405
8. Ministry of Health Viet Nam. National guidelines for reproductive healthcare, 2016.
9. World Health Organization. WHO antenatal care randomized trial: manual for the implementation of the new model. Geneva: World Health Organization, 2002.
10. UNICEF and General Statistics Office. Viet Nam Multiple Indiciator Cluster Survey 2014, Final Report. Ha Noi, Viet Nam, 2015.
11. UNFPA. Improvning the Quality of Reproductive Health Care Services in Viet Nam. The Role of National Standards and Guidelines for Reproductive Health Care Services. Ha Noi, 2007.
12. Gammeltoft T, Nguyen HT. The commodification of obstetric ultrasound scanning in Hanoi, Viet Nam. *Reprod Health Matters* 2007;15(29):163-71. doi: 10.1016/S0968-8080(06)29280-2 [published Online First: 2007/05/22]
13. Vujicic M, Shengelia B, Alfano M, et al. Physician shortages in rural Vietnam: using a labor market approach to inform policy. *Soc Sci Med* 2011;73(7):970-7. doi: 10.1016/j.socscimed.2011.06.010
14. Tran TK, Nguyen CTK, Nguyen HD, et al. Urban - rural disparities in antenatal care utilization: a study of two cohorts of pregnant women in Vietnam. *BMC health services research* 2011;11:120-20. doi: 10.1186/1472-6963-11-120
15. The World Bank, Vietnam. http://data.worldbank.org, 2017.
16. WHO and the Ministry of Health Viet Nam. Health Service Delivery Profile Viet Nam, 2012.
17. Ministry of Health, Viet Nam. Department of Planning and Finance and Health Insurance. Social health insurance scheme in Vietnam. Achievements & Challanges. Tokyo, 2016.
18. Raven J, Liu X, Hu D, et al. Improving neonatal health in remote rural areas in China and Vietnam: synthesis report. Liverpool School of Tropical Medicine, Liverpool UK, 2015
19. Ahman A, Kidanto HL, Ngarina M, et al. 'Essential but not always available when needed' - an interview study of physicians' experiences and views regarding use of obstetric ultrasound in Tanzania. *Glob Health Action* 2016;9(1):31062. doi: 10.3402/gha.v9.31062

For peer review only

20. Edvardsson K, Ntaganira J, Ahman A, et al. Physicians' experiences and views on the role of obstetric ultrasound in rural and urban Rwanda: a qualitative study. *Trop Med Int Health* 2016;21(7):895-906. doi: 10.1111/tmi.12718

21. Holmlund S, Ntaganira J, Edvardsson K, et al. Improved maternity care if midwives learn to perform ultrasound: a qualitative study of Rwandan midwives' experiences and views of obstetric ultrasound. *Glob Health Action* 2017;10(1):1350451. doi: 10.1080/16549716.2017.1350451

22. Åhman A, Edvardsson K, Lesio Kidanto H, et al. ‘Without ultrasound you can’t reach the best decision’ – Midwives’ experiences and views of the role of ultrasound in maternity care in Dar Es Salaam, Tanzania. *Sexual & Reproductive Healthcare* 2018;15:28-34. doi: https://doi.org/10.1016/j.srhc.2017.11.007

23. Edvardsson K, Small R, Persson M, et al. 'Ultrasound is an invaluable third eye, but it can't see everything': a qualitative study with obstetricians in Australia. *BMC Pregnancy Childbirth* 2014;14:363. doi: 10.1186/1471-2393-14-363

24. Ahman A, Persson M, Edvardsson K, et al. Two sides of the same coin--an interview study of Swedish obstetricians' experiences using ultrasound in pregnancy management. *BMC Pregnancy Childbirth* 2015;15:304. doi: 10.1186/s12884-015-0743-5

25. Edvardsson K, Mogren I, Lalos A, et al. A routine tool with far-reaching influence: Australian midwives' views on the use of ultrasound during pregnancy. *BMC Pregnancy Childbirth* 2015;15:195. doi: 10.1186/s12884-015-0632-y

26. Edvardsson K, Lalos A, Ahman A, et al. Increasing possibilities - Increasing dilemmas: A qualitative study of Swedish midwives' experiences of ultrasound use in pregnancy. *Midwifery* 2016;42:46-53. doi: 10.1016/j.midw.2016.09.009

27. Edvardsson K, Small R, Lalos A, et al. Ultrasound's 'window on the womb' brings ethical challenges for balancing maternal and fetal health interests: obstetricians' experiences in Australia. *BMC Med Ethics* 2015;16:31. doi: 10.1186/s12910-015-0023-y

28. Kongnyuy EJ, van den Broek N. The use of ultrasonography in obstetrics in developing countries. *Trop Doct* 2007;37(2):70-2. doi: 10.1177/004947550703700203

29. Holmlund S, Ntaganira J, Edvardsson K, et al. Health professionals’ experiences and views on obstetric ultrasound in Rwanda: A cross-sectional study. *PLOS ONE* 2018;13(12):e0208387. doi: 10.1371/journal.pone.0208387

30. Edvardsson K, Åhman A, Fagerli TA, et al. Norwegian obstetricians’ experiences of the use of ultrasound in pregnancy management. A qualitative study. *Sexual & Reproductive Healthcare* 2018;15:69-76. doi: https://doi.org/10.1016/j.srhc.2017.12.001

31. Edvardsson K, Graner S, Thi LP, et al. 'Women think pregnancy management means obstetric ultrasound': Vietnamese obstetricians' views on the use of ultrasound during pregnancy. *Glob Health Action* 2015;8:28405. doi: 10.3402/gha.v8.28405

32. Gonzaga MA, Kiguli-Malwadde E, Businge F, et al. Utilisation of obstetric sonography at a peri-urban health centre in Uganda. *The Pan African Medical Journal* 2010;7:24.

33. Bricker L, Medley N, Pratt JJ. Routine ultrasound in late pregnancy (after 24 weeks' gestation). *Cochrane Database of Systematic Reviews* 2015(6) doi: 10.1002/14651858.CD001451.pub4

34. Garcia J, Bricker L, Henderson J, et al. Women's views of pregnancy ultrasound: a systematic review. *Birth* 2002;29(4):225-50. [published Online First: 2002/11/15]

35. Whitworth M, Bricker L, Mullan C. Ultrasound for fetal assessment in early pregnancy. *Cochrane Database Syst Rev* 2015(7):CD007058. doi: 10.1002/14651858.CD007058.pub3

36. Mensah YB, Nkyekyer K, Mensah K. The Ghanaian Woman's Experience and Perception of Ultrasound Use in Antenatal Care. *Ghana Medical Journal* 2014;48(1):31-38.

For peer review only - http://bmjopen.bmj.com/site/about/guidelines.xhtml

37. Sippel S, Muruganandan K, Levine A, et al. Review article: Use of ultrasound in the developing world. *International Journal of Emergency Medicine* 2011;4:72-72. doi: 10.1186/1865-1380-4-72
38. Van Minh H, Oh J, Giang KB, et al. Multiple vulnerabilities and maternal healthcare in Vietnam: findings from the Multiple Indicator Cluster Surveys, 2000, 2006, and 2011. *Global Health Action* 2016;9:10.3402/gha.v9.29386. doi: 10.3402/gha.v9.29386
39. Malqvist M, Lincetto O, Du NH, et al. Maternal health care utilization in Viet Nam: increasing ethnic inequity. *Bull World Health Organ* 2013;91(4):254-61. doi: 10.2471/BLT.12.112425
40. Gammeltoft T, Nguyen HT. Fetal conditions and fatal decisions: ethical dilemmas in ultrasound screening in Vietnam. *Soc Sci Med* 2007;64(11):2248-59. doi: 10.1016/j.socscimed.2007.02.015
41. Howe D. Ethics of prenatal ultrasound. *Best Practice & Research Clinical Obstetrics & Gynaecology* 2014;28(3):443-51. doi: https://doi.org/10.1016/j.bpobgyn.2013.10.005
42. Shah S, Bellows BA, Adedipe AA, et al. Perceived barriers in the use of ultrasound in developing countries. *Critical Ultrasound Journal* 2015;7(1):11. doi: 10.1186/s13089-015-0028-2
43. LaGrone LN, Sadasivam V, Kushner AL, et al. A review of training opportunities for ultrasonography in low and middle income countries. *Trop Med Int Health* 2012;17(7):808-19. doi: 10.1111/j.1365-3156.2012.03014.x
44. Swanson JO, Kawooya MG, Swanson DL, et al. The diagnostic impact of limited, screening obstetric ultrasound when performed by midwives in rural Uganda. *J Perinatol* 2014;34(7):508-12. doi: 10.1038/jp.2014.54 [published Online First: 2014/04/05]
45. Nathan RO, Swanson JO, Swanson DL, et al. Evaluation of Focused Obstetric Ultrasound Examinations by Health Care Personnel in the Democratic Republic of Congo, Guatemala, Kenya, Pakistan, and Zambia. *Current Problems in Diagnostic Radiology* 2017;46(3):210-15. doi: https://doi.org/10.1067/j.cpradiol.2016.11.001
46. Bentley S, Hexom B, Nelson BP. Evaluation of an Obstetric Ultrasound Curriculum for Midwives in Liberia. *J Ultrasound Med* 2015;34(9):1563-8. doi: 10.7863/ultra.15.14.08017
47. Nancy Waxler-Morrison JMA, Elisabeth Richardson, Natalie A. Chambers. Cross-Cultural Caring: A handbook for Health Professionals. 2nd ed. Vancouver, Canada: UBC Press 2011.
48. Jones PS, O'Toole MT, Hoa N, et al. Empowerment of nursing as a socially significant profession in Vietnam. *Journal of Nursing Scholarship* 2000;32(3):317-21. doi: 10.1111/j.1547-5069.2000.00317.x
49. The World Bank. World Health Organization's Global Health Workforce Statistics, OECD, supplemented by country data 2013 [cited 2017 2017-01.15]. Available from: www.data.worldbank.org.
50. World Health Organization. Regional Office for the Western Pacific. Human resources for health: action framework for the Western Pacific Region (2011-2015). Manila, 2012.

For peer review only

779 # Figure legend

780

781 **Fig. 1. Obstetricians/Gynaecologists' self-rated skills for specified ultrasound**
782 **examinations (n=227).** Reported skill levels are presented with proportions

For peer review only

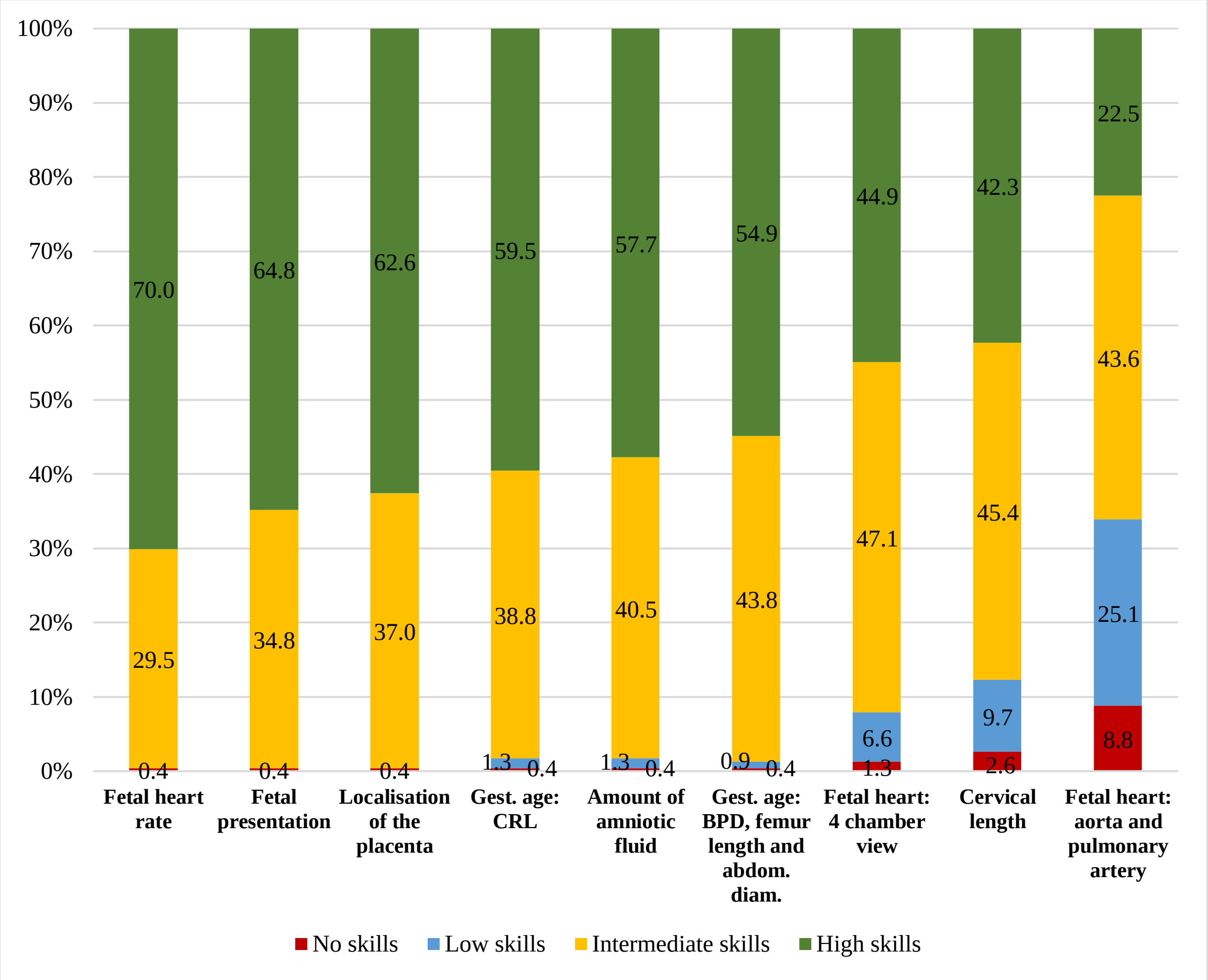
100%
90%
80%
70%
60%
50%
40%
30%
20%
10%
0%
70.0
29.5
0.4
64.8
34.8
0.4
62.6
37.0
0.4
59.5
38.8
1.3
0.4
57.7
40.5
1.3
0.4
54.9
43.8
0.9
0.4
44.9
47.1
6.6
1.3
42.3
45.4
9.7
2.6
22.5
43.6
25.1
8.8
Fetal heart rate
Fetal presentation
Localisation of the placenta
Gest. age: CRL
Amount of amniotic fluid
Gest. age: BPD, femur length and abdom. diam.
Fetal heart: 4 chamber view
Cervical length
Fetal heart: aorta and pulmonary artery
No skills
Low skills
Intermediate skills
High skills

STROBE 2007 (v4) Statement—Checklist of items that should be included in reports of *cross-sectional studies*

Section/Topic	Item #	Recommendation	Reported on page #
**Title and abstract**	1	(*a*) Indicate the study's design with a commonly used term in the title or the abstract	1
		(*b*) Provide in the abstract an informative and balanced summary of what was done and what was found	3-4
**Introduction**			
Background/rationale	2	Explain the scientific background and rationale for the investigation being reported	6-7
Objectives	3	State specific objectives, including any prespecified hypotheses	6-7
**Methods**			
Study design	4	Present key elements of study design early in the paper	9
Setting	5	Describe the setting, locations, and relevant dates, including periods of recruitment, exposure, follow-up, and data collection	8-11
Participants	6	(*a*) Give the eligibility criteria, and the sources and methods of selection of participants	10
Variables	7	Clearly define all outcomes, exposures, predictors, potential confounders, and effect modifiers. Give diagnostic criteria, if applicable	11-13
Data sources/ measurement	8*	For each variable of interest, give sources of data and details of methods of assessment (measurement). Describe comparability of assessment methods if there is more than one group	13
Bias	9	Describe any efforts to address potential sources of bias	9-10
Study size	10	Explain how the study size was arrived at	9
Quantitative variables	11	Explain how quantitative variables were handled in the analyses. If applicable, describe which groupings were chosen and why	11-13
Statistical methods	12	(*a*) Describe all statistical methods, including those used to control for confounding	13
		(*b*) Describe any methods used to examine subgroups and interactions	
		(*c*) Explain how missing data were addressed	
		(*d*) If applicable, describe analytical methods taking account of sampling strategy	
		(*e*) Describe any sensitivity analyses	
**Results**			

For peer review only

Participants	13*	(a) Report numbers of individuals at each stage of study—eg numbers potentially eligible, examined for eligibility, confirmed eligible, included in the study, completing follow-up, and analysed	14-15
		(b) Give reasons for non-participation at each stage	10
		(c) Consider use of a flow diagram	
Descriptive data	14*	(a) Give characteristics of study participants (eg demographic, clinical, social) and information on exposures and potential confounders	14-15
		(b) Indicate number of participants with missing data for each variable of interest	12, 14-19
Outcome data	15*	Report numbers of outcome events or summary measures	14-21
Main results	16	(*a*) Give unadjusted estimates and, if applicable, confounder-adjusted estimates and their precision (eg, 95% confidence interval). Make clear which confounders were adjusted for and why they were included	14-21
		(*b*) Report category boundaries when continuous variables were categorized	
		(*c*) If relevant, consider translating estimates of relative risk into absolute risk for a meaningful time period	
Other analyses	17	Report other analyses done—eg analyses of subgroups and interactions, and sensitivity analyses	
**Discussion**			
Key results	18	Summarise key results with reference to study objectives	22, 27
Limitations	19	Discuss limitations of the study, taking into account sources of potential bias or imprecision. Discuss both direction and magnitude of any potential bias	26-27
Interpretation	20	Give a cautious overall interpretation of results considering objectives, limitations, multiplicity of analyses, results from similar studies, and other relevant evidence	22-26
Generalisability	21	Discuss the generalisability (external validity) of the study results	26-27
**Other information**			
Funding	22	Give the source of funding and the role of the funders for the present study and, if applicable, for the original study on which the present article is based	29

*Give information separately for cases and controls in case-control studies and, if applicable, for exposed and unexposed groups in cohort and cross-sectional studies.

Note: An Explanation and Elaboration article discusses each checklist item and gives methodological background and published examples of transparent reporting. The STROBE checklist is best used in conjunction with this article (freely available on the Web sites of PLoS Medicine at http://www.plosmedicine.org/, Annals of Internal Medicine at http://www.annals.org/, and Epidemiology at http://www.epidem.com/). Information on the STROBE Initiative is available at www.strobe-statement.org.

For peer review only

BMJ Open

Health professionals' experiences and views on obstetric ultrasound in Vietnam: a regional, cross-sectional study

Journal:	*BMJ Open*
Manuscript ID	bmjopen-2019-031761.R2
Article Type:	Original research
Date Submitted by the Author:	26-Aug-2019

Complete List of Authors:	Holmlund, Sophia; Umea Universitet Medicinska fakulteten, Clinical Sciences, Obstetrics and Gynecology<br>Pham Thi, Lan; Hanoi Medical University, Department of Dermatology and Venereology<br>Edvardsson, Kristina; Umea Universitet Medicinska fakulteten, Department of Clinical Sciences, Obstetrics and Gynecology; La Trobe University, Judith Lumley Centre, School of Nursing and Midwifery<br>Ho Dang, Phuc ; Vietnam Academy of Science and Technology, Department of Probability and Mathematical Statistics, Institute of Mathematics<br>Ntaganira, Joseph; University of Rwanda College of Medicine and Health Sciences, School of Public Health<br>Small, Rhonda; La Trobe University, Judith Lumley Centre, School of Nursing and Midwifery; Karolinska Institutet, Department of Women's and Children's and Reproductive Health<br>Lesio Kidanto, Hussein; Aga Khan University - Tanzania, Department of Obstetrics and Gynecology<br>Ngarina, Matilda; Muhimbili National Hospital, Department of Obstetrics and Gynecology<br>Mogren, Ingrid; Umeå Universitet Medicinska fakulteten, Department of Clinical Sciences, Obstetrics and Gynecology; La Trobe University, Judith Lumley Centre, School of Nursing and Midwifery

**Primary Subject Heading**:	Obstetrics and gynaecology
Secondary Subject Heading:	Epidemiology, Medical education and training, Public health, Reproductive medicine
Keywords:	prenatal ultrasonography, obstetricians, midwives, prenatal care, pregnancy, Vietnam

SCHOLARONE™
Manuscripts

Health professionals' experiences and views on obstetric ultrasound in Vietnam: a regional, cross-sectional study

Sophia Holmlund[1*], Lan Pham Thi[2], Kristina Edvardsson[1,3], Phuc Ho Dang[4], Joseph Ntaganira[5], Rhonda Small[3,6], Hussein Lesio Kidanto[7], Matilda Ngarina[8], Ingrid Mogren[1,3]

[1]Department of Clinical Sciences, Obstetrics and Gynecology, Umeå University, Umeå, Sweden

[2]Department of Dermatology and Venereology, Hanoi Medical University, Hanoi, Vietnam

[3]Judith Lumley Centre, School of Nursing and Midwifery, La Trobe University, Melbourne, Australia

[4]Department of Probability and Mathematical Statistics, Institute of Mathematics, Vietnam Academy of Science and Technology, Hanoi, Vietnam

[5]School of Public Health, College of Medicine and Health Sciences, University of Rwanda, Kigali, Rwanda

[6]Department of Women's and Children's and Reproductive Health, Karolinska Institutet, Stockholm, Sweden

[7]Department of Obstetrics and Gynecology, Aga Khan University, Dar es Salaam, Tanzania

[8]Department of Obstetrics and Gynecology, Muhimbili National Hospital, Dar es Salaam, Tanzania

Email addresses:

SH: sofia.holmlund@umu.se

LPT: lanphamthi009@gmail.com

KE: k.edvardsson@latrobe.edu.au

PHD: hodang54@yahoo.com

JN: jntaganira@ur.ac.rw

RS: r.small@latrobe.edu.au

HLK: hkidanto@gmail.com

MN: mmatty71@gmail.com

IM: ingrid.mogren@umu.se

[*]Corresponding author

For peer review only

Address: Department of Clinical Sciences, Obstetrics and Gynecology, Umeå University, SE 901 87 Umeå, Sweden, sofia.holmlund@umu.se, +46 90-7850463

For peer review only

Abstract

Objectives

Obstetric ultrasound is an important part of antenatal care in Vietnam, although there are great differences in access to antenatal care and ultrasound services across the country. The aim of this study was to explore Vietnamese health professionals' experiences and views of obstetric ultrasound in relation to clinical management, resources and skills.

Design

A cross-sectional questionnaire study was performed as part of the **CRO**ss **C**ountry **U**ltra**S**ound study (CROCUS study).

Setting

Health facilities (N=29) in urban, semi-urban and rural areas of Hanoi region, Vietnam.

Participants

Participants were 289 obstetricians/gynaecologists and 535 midwives.

Results

A majority (88%) of participants agreed that "every woman should undergo ultrasound examination" in pregnancy to determine gestational age. Participants reported an average of six ultrasound examinations as medically indicated during an uncomplicated pregnancy.

For peer review only

Access to ultrasound at participants' workplaces was reported as good regardless of health facility level. Most participants performing ultrasound reported high level skills for fetal heart rate examination (70%), whereas few (23%) reported being skilled in examination of the anatomy of the fetal heart. Insufficient ultrasound training leading to suboptimal pregnancy management was reported by 37% of all participants. "Better quality of ultrasound machines", "more physicians trained in ultrasound" and "more training for health professionals currently performing ultrasound" were reported as ways to improve the utilisation of ultrasound.

Conclusions

Obstetric ultrasound is used as an integral part of antenatal care at all selected health facility levels in the region of Hanoi, and access was reported as high. However, reports of insufficient ultrasound training resulting in suboptimal pregnancy management indicates a need for additional training of ultrasound operators to improve utilisation of ultrasound.

Strengths and limitations of this study

- The study questionnaire was developed based on the results from earlier qualitative studies performed with obstetricians/physicians and midwives/nurses in six different countries (the CROCUS study).
- The strengths of this study include participants of different health professional categories recruited from different levels of the health care system in urban, semi-urban and rural areas of Hanoi.
- The research team comprised two Vietnamese researchers familiar with the setting and the health care system which strengthens the interpretation of data.

For peer review only

- One limitation of this study may be the translation of the questionnaire from English to Vietnamese, however, measures to reduce the risk of losing the intended meaning of questions and statements were implemented.
- Since previous studies within this research domain are lacking, the power calculation was based on assumptions of proportions for one outcome variable in relation to one background variable, and may therefore mean uncertainty of the required study sample.

Keywords

prenatal ultrasonography; health personnel; obstetricians; midwives; prenatal care; pregnancy; Vietnam

For peer review only

Background

Sufficient antenatal care (ANC) services and skilled birth attendance are important factors contributing to safer deliveries, reductions in obstetric complications, and decreased maternal and neonatal morbidity and mortality [1]. Timely and appropriate evidence-based practices in ANC including health promotion, screening and diagnosis, and prevention for diseases can save lives [2]. Since 2016, the World Health Organization (WHO) recommends a minimum of eight ANC contacts during pregnancy and one ultrasound examination before 24 weeks of gestation. The aim of the recommendation of an early ultrasound scan is to estimate gestational age, improve detection of multiple pregnancy and fetal anomaly, reduce induction of post-term pregnancy and improve women's pregnancy experience [2]. Ultrasound is an important part of ANC in high income countries [3], and clinical trials show that ultrasound may improve management and pregnancy outcomes in the developing world [4].

The utilisation of reproductive health services in Vietnam has substantially increased over recent decades, but there are still inequities in the country [5]. Low education, poverty, ethnic minority status [6] and living in rural areas are factors associated with decreased access to reproductive health services [5]. Ultrasound has become a central tool in ANC services in Vietnam [7]. Currently the Ministry of Health in Vietnam recommends at least four ANC visits [8], in accordance with the previous recommendation by WHO [9]. Additionally, three ultrasound examinations are recommended during pregnancy, in gestational week 11-13, 20-24 and 30-32 [8]. In 2014, more than 70% of pregnant women in Vietnam received at least four ANC visits [10]. However, almost half of these women reported not having urine or blood samples taken or blood pressure measurement done during their last pregnancy [10]. The private health sector is a continuously growing part of health care services in Vietnam, especially for provision of legal abortion services and obstetric ultrasound examinations [11]. Limited salary

For peer review only

for physicians creates incentives for both public and private practitioners to search for additional income through provision of these services [12 13]. Commercialisation of ultrasound services has led to urban Vietnamese women having an average of more than six ultrasound scans during pregnancy which is a high number of examinations from an international perspective [12 14].

Study rationale

Few studies have investigated the use of obstetric ultrasound in Vietnam from health professionals' perspectives, although ultrasound is frequently used during pregnancy. This study serves to fill a knowledge gap and can benefit authorities in their work to further develop education and improvement of guidelines on obstetric ultrasound use. This study is part of the **CRO**ss **C**ountry **U**ltra**S**ound study (CROCUS) investigating health professionals' experiences and views of the use of ultrasound in high-, middle and low-resource countries.

Aims

The overall aim of this study was to explore different aspects of obstetric ultrasound in Vietnam from health professionals' perspectives.

The research questions investigated were:

- What are health professionals' views of the role of obstetric ultrasound for clinical management of pregnancy?
- How do health professionals view access to obstetric ultrasound?
- How do health professionals assess their skills in performing obstetric ultrasound examinations?

For peer review only

- What do health professionals believe could improve the utilisation of obstetric ultrasound?

Materials and methods

The Vietnamese setting

Since 2010, Vietnam has been classified as a lower middle income country and has undergone substantial economic development in recent decades [1]. The maternal mortality rate has decreased from 139/100 000 in 1990 to 54/100 000 live births in 2015 [15]. Most inpatient health care is provided by public hospitals but for outpatient care, private clinics account for a large number of patients [16]. Vietnam is divided into 63 provinces, 698 districts and 11,121 communes [17]. In each commune, a village health worker (VHW) provides health promotion, immunisation, nutrition services and attends births in remote areas [18]. At the community health centre level (CHS), a midwife or an assistant doctor is in charge of maternal health services and provides ANC, assists normal delivery, and provides postnatal care, immunisation services and supervision of the VHW. At district level, ANC, delivery care including caesarean sections and newborn care are provided at hospitals [18] while maternity homes deliver basic prenatal and delivery services [16 18]. Provincial hospitals provide more specialised health care, and referrals from lower health care levels to provincial level are undertaken if complications occur during pregnancy or delivery [18]. At the top of the health care system there are several national hospitals providing specialised care and receiving referrals from lower levels [16].

Study design

For peer review only

This cross-sectional study used a questionnaire to investigate a number of research questions related to obstetric ultrasound with obstetricians/gynaecologists and midwives providing pregnancy, delivery and postpartum care to women in the region of Hanoi, Vietnam.

Sampling

Owing to the lack of findings from similar studies, a sample size of 290 obstetricians/gynaecologists and a corresponding number of midwives (n=290) was calculated based on plausible estimations of prevalence of background characteristics and outcome variables. The calculation was based on the outcome requiring the largest sample size "every woman should undergo ultrasound examination in pregnancy to determine gestational age" and the background variable "work experience over and under 5 years", to detect a difference in proportion of 0.10 with a power of 80% and a significance level of 5%.

Purposive sampling was used to obtain a representative sample of health professionals caring for pregnant women at different levels of health facilities in urban, semi-urban and rural areas in the region of Hanoi. One national hospital, one provincial hospital, 24 district hospitals and three maternity homes were included in the study, in total 29 health facilities.

Questionnaire

The study questionnaire was developed based on the results from the earlier qualitative studies performed in the CROCUS study [7 19-27]. Sociodemographic characteristics, evaluation of self-reported skills in performing ultrasound and questions about access to obstetric ultrasound and health professionals' views on what may improve utilisation of ultrasound in Vietnam were included, among other items. The questionnaire included both questions and statements, and the items had either fixed or Likert-scale response options. This analysis investigates the research questions noted above, utilising 45 of the 105 questions and

For peer review only - http://bmjopen.bmj.com/site/about/guidelines.xhtml

statements. The questionnaire was developed first in English and thereafter translated to Vietnamese by a native Vietnamese speaker independent of the research team. The Vietnamese version of the questionnaire was also back-translated to English by another external person. This check resulted in minor adjustments of some words, but demonstrated that the Vietnamese translation had retained the overall meaning of the English. The questionnaire was pilot-tested with 10 obstetricians, 6 midwives and 2 sonographers. No further revisions of the questionnaire were required as a result of piloting.

Data collection procedures including recruitment of study participants

The data collection was performed in April 2017 by four experienced data collectors supervised by two Vietnamese senior researchers in the research team. Before the start of the data collection, data collectors were trained by the research team and all questions and statements in the questionnaire were discussed to ensure correct understanding. The two Vietnamese researchers initiated contact with the directors of all selected health facilities and all of them agreed to assist with recruitment of participants. For this study, we aimed to include health professionals caring for pregnant women and with different experiences in relation to use of obstetric ultrasound. Eligible participants were health professionals managing pregnant women at the maternity wards on the day of data collection at each study site. No eligible participant declined participation in the study. The primary sample included 890 participants. Six individuals working as radiology technicians were excluded from the primary sample as the they did not fulfil the inclusion criteria, and finally 60 sonographers were also excluded from the primary sample since they constituted a small part of the sample, and further did not contribute to clinical management after their obstetric ultrasound examination. The final sample (N=824) included the following health professionals:

For peer review only

obstetricians/gynaecologists (n=289) and midwives (n=535). Participation was anonymous and all questionnaires were given a unique code. Safe storage of questionnaires was undertaken in accordance with national procedures and regulations. Data were entered into an SPSS file at the Hanoi Medical University, by two experienced data clerks. To evaluate the quality of the data entry, every 10th questionnaire based on the number order of identification codes, was selected for data re-entry. The data from all 107 variables in 89 questionnaires were re-entered in the SPSS file by the first author. The rate of error was 1.4 %. The identified errors in the SPSS file were corrected.

Patient and Public Involvement

This research was done without patient involvement.

Independent variables

Age was calculated as a continuous variable using birth year and year of data collection. For some analyses age was dichotomised as age 34 years or less and 35 years and above. *Gender* included female or male. *Health profession* included the following response options in the questionnaire: obstetrician/gynaecologist, general practitioner, resident physician, physician other (please specify), midwife, radiologist/sonographer and "other" (please specify). *Health profession* was thereafter categorised into two groups: obstetricians/gynaecologists and midwives. Resident physicians undergoing post-graduate training (n=9) and general practitioners (n=12) were also included in the category obstetricians/gynaecologists because they worked at the same department and performed similar work tasks as the obstetricians/gynaecologists. One participant who was an anaesthesiologist by profession, but was working with maternity care, was categorised as an obstetrician/gynaecologist. One nurse working in maternity care was categorised as a midwife. *Health facilities* included the

For peer review only - http://bmjopen.bmj.com/site/about/guidelines.xhtml

response options national hospital, provincial hospital, district hospital and maternity home. The variable *health facilities* was dichotomised into national hospital/provincial hospital and district hospital/maternity home in some analyses. *Area of health facility* was categorised as hospitals in urban (n=7), semi-urban (n=5) and rural (n=17) areas of Hanoi. *Type of health care* was classified as public, private and both public and private health care. No participant reported working only in private health care. *Number of ultrasound examinations indicated in an uncomplicated pregnancy* was categorised based on the three recommended number of ultrasound examinations by the Ministry of Health in Vietnam; 3 examinations or less and 4 examinations or more.

Dependent variables

The dependent variables with fixed response alternatives are presented in Table 1. For the statements related to “the role of ultrasound for clinical management” and “resources and training of obstetric ultrasound”, the response options were dichotomised into *disagree* or *strongly disagree* and *agree* or *strongly agree* in logistic regression analyses. The question “do you have a role in decision-making regarding clinical management on the basis of obstetric ultrasound examinations” was both used as an independent and dependent variable and the response options were dichotomised into *no* and *yes* for some analyses. The response options of the statements related to “improving utilisation of ultrasound” were categorised as *not at all* or *not very much* and *a fair amount* or *a great deal* in logistic regression analyses. The response option *neutral* or *don´t know* was not included in either of these categories. For most statements, the response options *neutral* and *don´t know* were selected by a small proportion of the participants.

Table 1. Questions or statements and their response options in the questionnaire

How often do you perform obstetric ultrasound examinations? [a]

For peer review only

How do you rate your skills in ultrasound in relation to the assessment/evaluation of:

- Fetal presentation[b]
- Localisation of the placenta[b]
- Fetal heart rate[b]
- Amniotic fluid amount[b]
- Gestational age estimated by CRL (crown-rump-length)[b]
- Gestational age estimated by biparietal diameter, femur length and abdominal diameter[b]
- Cervical length[b]
- Fetal heart: 4 chamber view[b]
- Fetal heart: aorta and pulmonary artery[b]
- Doppler: umbilical artery[b]

Do you have a role in decision-making regarding clinical management on the basis of obstetric ultrasound examinations?[c]

How often do you make decisions based on the results from obstetric ultrasound examinations in your clinical work? [a]

What do you believe would improve the utilisation of ultrasound at your clinic/work place?

- More ultrasound machines[d]
- Better quality of ultrasound machines[d]
- More training for health professionals currently performing ultrasound[d]
- More doctors trained in ultrasound[d]
- (More) midwives trained in ultrasound[d]

Statements on ultrasound resources and training

- Pregnant women in my country have access to dating ultrasound (i.e. estimation of gestational age)[e]
- Pregnant women in my country have access to fetal anomaly screening[e]
- Pregnant women in my country have access to obstetric ultrasound independent of area of living[e]
- Pregnant women in my country have access to obsteric ultrasound independent of income[e]
- There are enough resources in my country to provide *medically indicated* obstetric ultrasound examinations to pregnant women who need it[e]
- At my workplace, there is always access to obstetric ultrasound when it is needed[e]
- At my workplace, lack of ultrasound training of the ultrasound operator sometimes leads to suboptimal pregnancy management[e]
- Maternity care in my country would improve if midwives were qualified to perform basic ultrasound examinations[e]

Statements on the role of ultrasound in clinical management of pregnancy

- Ultrasound is decisive in pregnancy management[e]
- Every woman should undergo ultrasound examination in pregnancy to determine gestational age[e]
- It is irresponsible of a pregnant woman to decline a dating scan[e]
- Ultrasound is safe to use for the pregnant woman and the fetus irrespective of the number of examinations[e]
- Ultrasound is important for expectant parents to bond with their fetus during pregnancy[e]

[a]Response options: Never, On a daily basis, On a weekly basis, On a monthly basis, More seldom than on a monthly basis

[b]Response options: No skills, Skill-level low, Skill-level intermediate, Skill-level high

[c]Response options: No, Yes a minor role, Yes a moderate role, Yes a major role

[d]Response options: Not at all, Not very much, A fair amount, A great deal, Don't know

[e]Response options: Strongly agree, Agree, Neutral, Disagree, Strongly disagree

Statistical analysis

For categorical variables, frequencies and percentages were analysed and Pearson's Chi-Square test was used for test of difference, with the level of significance set at $p<0.05$. For continuous variables, mean values and their standard deviations (SD) were presented. Univariable and multivariable logistic regression was undertaken and are presented with odds ratios (OR) and their 95% confidence intervals (CI). The independent and dependent variables

For peer review only

used for logistic regression are reported in their specific sections above as well as in Table 1. All independent variables were entered into the univariable logistic regression analysis, however only the statistically significant variables were included in the final multivariable logistic regression models. Statistical analyses were performed using SPSS version 23.

Results

Background characteristics of the study sample

A total of 824 participants aged 21-60 years (mean age 34.8 years) were enrolled in the study. The distribution of health professionals was obstetricians/gynaecologists (35.1%), and midwives (64.9%; Table 2). One third of the participants (28.1%) was performing obstetric ultrasound and mainly on a daily basis (66.5%). All obstetricians/gynaecologists working in maternity homes (100%) were performing ultrasound as well as a majority of obstetricians/gynaecologists working in provincial hospitals (84.9%), district hospitals (75.2%) and national hospitals (75.7%). The mean estimated number of ultrasound examinations per day was 15.7 (median 10, range 1-100) for obstetricians/gynaecologists. Obstetricians/gynaecologists performing more than ten examinations per day were significantly older (≥35 years) than those obstetricians/gynaecologists performing ten or fewer examinations per day (p<0.001). A few participants at all health care levels (8.3%) reported that midwives performed ultrasound in their workplace. Participants at district hospitals (7.0%) and maternity homes (6.3%) reported the lowest percentage of midwives performing ultrasound in their workplace.

For peer review only

327 **Table 2.** Background characteristics of the study sample (N=824)
328

Variable	All health professionals Total= 824 n (%)	Obstetricians/Gynaecologists Total=289 n (%)	Midwives Total=535 n (%)
**Age (years)**	***811 (98.4)***	***286 (99.0)***	***525 (98.1)***
Mean; SD[a]	34.8; 8.7	36.6; 9.2	33.7; 8.3
Min-Max	21-60	23-60	21-55
**Years in profession**	***818 (99.3)***	***288 (99.7)***	***530 (99.1)***
Mean; SD[a]	10.5; 8.3	10.4; 8.9	10.5; 8.1
Min-max	0-35	0-32	0.5-35
**Years in health care**	***817 (99.2)***	***287 (99.3)***	***530 (99.1)***
Mean; SD[a]	11.1; 8.5	11.6; 9.2	10.9; 8.2
Min-max	0-38	0-38	0.5-35
**Gender**	***824 (100)***	***289 (100)***	***535 (100)***
Male	123 (14.9)	123 (42.6)	0
Female	701 (85.1)	166 (57.4)	535 (100.0)
**Marital status**	***817 (99.2)***	***287 (99.3)***	***530 (99.1)***
Married	714 (87.4)	242 (84.3)	472 (89.1)
Separated/Divorced	1 (0.1)	0 (0.0)	1 (0.2)
Widowed	4 (0.5)	2 (0.7)	2 (0.4)
Not married/Single	98 (12.0)	43 (15.0)	55 (10.4)
**Having children**	***821 (99.6)***	***288 (99.7)***	***533 (99.6)***
Yes	684 (83.3)	230 (79.9)	454 (85.2)
No	137 (16.7)	58 (20.1)	79 (14.8)
**Type of health care**	***823 (99.9)***	***289 (100)***	***534 (99.8)***
Public	789 (95.9)	268 (92.7)	521 (97.6)
Both public and private	34 (4.1)	21 (7.3)	13 (2.4)
**Level of health facility[b]**	***824 (100)***	***289 (100)***	***535 (100)***
National hospital	144 (17.5)	74 (25.6)	70 (13.1)
Provincial hospital	184 (22.3)	86 (29.8)	98 (18.3)
District hospital	464 (56.3)	121 (41.9)	343 (64.1)
Maternity home	32 (3.9)	8 (2.8)	24 (4.5)
**Area of health facility[c]**	***824 (100)***	***289 (100)***	***535 (100)***
Urban	439 (53.3)	191 (66.1)	248 (46.4)
Semi-urban	129 (15.7)	35 (12.1)	94 (17.6)
Rural	256 (31.1)	63 (21.8)	193 (36.1)
**Provision of maternity services[d]**			
Antenatal care	683 (83.0)	261 (90.3)	422 (79.0)
Intrapartum care	642 (78.0)	245 (84.8)	397 (74.3)
Postpartum care	688 (83.6)	235 (81.3)	453 (84.8)
Do not currently provide maternity care	32 (3.9)	10 (3.5)	23 (4.3)
**Performing ultrasound[e]**	***823 (99.9)***	***289 (100)***	***534 (99.8)***
Yes	231 (28.1)	228[f] (78.9)	3 (0.6)
No	592 (71.9)	61 (21.1)	531 (99.4)

329 [a]SD= Standard Deviation
330 [b]Number of participants at specified health facilities
331 [c]Number of participants at specified areas of health facilities
332 [d]Item in questionnaire: "Which of the following maternity services do you provide? (Please tick all that apply)"
333 [e]Performing obstetric ultrasound examinations
334 [f]One participant have not rated the skills in relation to different tasks during ultrasound examinations

For peer review only

The role of obstetric ultrasound

Most participants (66.2%) agreed or strongly agreed that "ultrasound is decisive in pregnancy management". Obstetricians/gynaecologists reported significantly lower agreement (55.7%) with the statement "ultrasound is decisive in pregnancy management" than midwives (71.8%; $p<0.001$). A majority (87.5%) of participants, independent of health profession, agreed or strongly agreed that "every woman should undergo ultrasound examination to determine gestational age". Most participants (75.0%) agreed or strongly agreed that "it is irresponsible of a pregnant woman to decline a dating ultrasound" and there was no significant difference in opinion between different health professionals. There was a significant difference in opinion between the different health professionals for the statement "ultrasound is safe to use for the woman and the fetus irrespective of the number of examinations", where obstetricians/gynaecologists were more likely to agree or strongly agree than midwives (OR 1.96; 95% CI 1.22-3.17). Participants reported an average of 5.9 ultrasound examinations as medically indicated during an uncomplicated pregnancy (obstetricians/gynaecologists: SD 2.7, range 2-15, and midwives: SD 2.6, range 2-20). A quarter of the ultrasound operators (25.1%) agreed with the national guidelines that three ultrasound examinations are medically indicated during an uncomplicated pregnancy. Midwives in comparison to obstetricians/gynaecologists, were more likely to agree or strongly agree that "ultrasound is important for expectant parents to bond with their fetus during pregnancy" (OR 1.59; 95% CI 1.13-2.26). The assessment that four ultrasound examinations or more are medically indicated in an uncomplicated pregnancy was associated with higher agreement with the statement "ultrasound is important for expectant parents to bond with their fetus during pregnancy", compared to those assessing that three ultrasound examinations or fewer are medically

For peer review only

indicated in a normal pregnancy (OR 1.61; 95% CI 1.03-2.50, adjusted for health profession and performing ultrasound or not).

Access to obstetric ultrasound

Most of the participants (95.6%-100%), regardless of health facility level, agreed or strongly agreed with the statement “there is always access to obstetric ultrasound when needed at my workplace”. Almost all participants (95.4%-100%) at all health facility levels reported that they agreed or strongly agreed with the statement “pregnant women in the country have access to dating ultrasound”. A majority of participants at all health facility levels (93.8%-95.1%), agreed or strongly agreed that “pregnant women in the country have access to fetal anomaly screening”. Midwives were more likely to agree or strongly agree that “pregnant women in the country have access to ultrasound independent of area of residence, compared with obstetricians/gynaecologists (OR 2.54; 95% CI 1.60-4.02). Participants in national hospitals and provincial hospitals reported significantly lower agreement with the statement “pregnant women in my country have access to ultrasound independent of income” than participants in district hospitals and maternity homes ($p<0.001$). Further results are presented in Table 3.

Table 3. Health professionals’ views on specified statements[a] (N=824)

Statement	Obstetricians/ Gynaecologists Total=289[c] n (%)	Midwives Total=535[c] n (%)	p-value[b]
**Pregnant women in my country have access to dating ultrasound (i.e. estimation of gestational age)**	***279 (96.5)***	***521 (97.4)***	0.98
Agree/Strongly agree	275 (98.6)	512 (98.3)	
Disagree/Strongly disagree	4 (1.4)	9 (1.7)	
**Pregnant women in my country have access to fetal anomaly screening**	***276 (95.5)***	***514 (96.1)***	0.16
Agree/Strongly agree	269 (97.5)	509 (99.0)	
Disagree/Strongly disagree	7 (2.5)	5 (1.0)	
**Pregnant women in my country have access to obstetric ultrasound independent of area of residence**	***248 (85.8)***	***485 (90.7)***	<0.001

For peer review only

Agree/Strongly agree	203 (81.9)	446 (92.0)	
Disagree/Strongly disagree	45 (18.1)	39 (8.0)	
**Pregnant women in my country have access to obstetric ultrasound independent of income**	***228 (78.9)***	***475 (88.8)***	0.082
Agree/Strongly agree	178 (78.1)	398 (83.8)	
Disagree/Strongly disagree	50 (21.9)	77 (16.2)	
**There are enough resources in my country to provide medically indicated obstetric ultrasound examinations to pregnant women who need it**	***234 (81.0)***	***447 (83.6)***	0.002
Agree/Strongly agree	185 (79.1)	395 (88.4)	
Disagree/Strongly disagree	49 (20.9)	52 (11.6)	
**At my workplace, there is always access to obstetric ultrasound when it is needed**	***282 (97.6)***	***519 (97.0)***	0.010
Agree/Strongly agree	272 (96.5)	515 (99.2)	
Disagree/Strongly disagree	10 (3.5)	4 (0.8)	
**At my workplace, lack of ultrasound training of the ultrasound operator sometimes leads to suboptimal pregnancy management**	***237 (82.0)***	***452 (84.5)***	0.086
Agree/Strongly agree	115 (48.5)	187 (41.4)	
Disagree/Strongly disagree	122 (51.5)	265 (58.6)	

[a]The response option *neutral* was excluded from analyses
[b]Pearsons Chi-Square Test with Yates' continuity correction for test of differences in proportion between the two groups of obstetricians/gynaecologists and midwives
[c]The number of participants included in each analysis are presented in relation to the total sample of each group of obstetricians/gynaecologists and midwives

Ultrasound operators' decision-making and self-rated skills

Almost all obstetricians/gynaecologists (92.2%) and a majority of midwives (59.4%) reported having a role in decision-making regarding clinical management on the basis of obstetric ultrasound examinations. There was a significant difference in the proportion of obstetricians/gynaecologists (38.6%) and midwives (20.5%) reporting having a major role in decision-making compared to having a minor or moderate role in decision-making ($p<0.001$). Approximately one third of all health professionals (37.1%) reported that they agreed or strongly agreed with the statement "at my workplace, lack of ultrasound training of the ultrasound operator sometimes leads to suboptimal pregnancy management". Health professionals performing ultrasound were asked to rate their skills in relation to different tasks during ultrasound examinations (Figure 1). For all the obstetricians/gynaecologists performing ultrasound (n=228), fetal heart rate was the examination where most participants

For peer review only

reported high skills (70.0%) and examination of the fetal heart: aorta and pulmonary artery was the examination with the lowest proportion of self-rated high skill level (22.5%).

Figure 1.

Improving utilisation of obstetric ultrasound

When health professionals were asked to assess how much particular strategies could improve the utilisation of ultrasound, “better quality of ultrasound machines” (94.0%), “more training for health professionals currently performing ultrasound” (92.8%) and “more physicians trained in ultrasound” (93.2%) were the statements with the highest numbers of participants reporting a fair amount or a great deal. Obstetricians/gynaecologists were more likely to agree that “more training for health professionals currently performing ultrasound would improve the utilisation of ultrasound” compared to midwives (OR 2.60; 95% CI 1.24-5.46). Further results are presented in Table 4. A majority of participants (69.0%) agreed or strongly agreed with the statement “maternity care in my country would improve if midwives were qualified to perform basic ultrasound examinations”, however midwives were more likely to agree or strongly agree than obstetricians/gynaecologists (OR 5.09; 95% CI 3.41-7.61). Participants working in rural hospitals (79.6%) and semi-urban hospitals (78.3%) were also more likely to agree with the previous statement than participants working in urban hospitals (60.0%) ($p<0.001$).

For peer review only

Table 4. Health professionals' views on factors that may improve utilisation of obstetric ultrasound, presented through preformed statements

**Variable**	**More ultrasound machines**[a]			**Better quality of ultrasound machines**[a]		
	Not at all or Not very much[b] **n (%)**	A fair amount or A great deal[b] **n (%)**	p-value[c]	Not at all or Not very much[b] **n (%)**	A fair amount or A great deal[b] **n (%)**	p-value[c]
**Health profession**	***149 (20.1)***	***593 (79.9)***	0.042	***40 (5.2)***	***732 (94.8)***	0.036
Obstetricians/Gynaecologists	65 (24.3)	203 (75.7)		8 (2.8)	276 (97.2)	
Midwives	84 (17.7)	390 (82.3)		32 (6.6)	456 (93.4)	
**Level of health facility**	***149 (20.1)***	***593 (79.9)***	0.003	***40 (5.2)***	***732 (94.8)***	0.232
National hospital	31 (24.4)	96 (75.6)		3 (2.2)	135 (97.8)	
Provincial hospital	43 (28.3)	109 (71.7)		12 (7.2)	154 (92.8)	
District hospital	73 (16.9)	359 (83.1)		24 (5.5)	413 (94.5)	
Maternity home	2 (6.5)	29 (93.5)		1 (3.2)	30 (96.8)	
**Performing ultrasound**[d]	***149 (20.1)***	***592 (79.9)***	0.045	***40 (5.2)***	***731 (94.8)***	0.060
Yes	53 (25.0)	159 (75.0)		6 (2.6)	221 (97.4)	
No	96 (18.1)	433 (81.9)		34 (6.3)	510 (93.8)	
	**More training for health professionals currently performing ultrasound**[a]			**More physicians trained in ultrasound**[a]		
	Not at all or Not very much[b] **n (%)**	A fair amount or A great deal[b] **n (%)**	p-value[c]	Not at all or Not very much[b] **n (%)**	A fair amount or A great deal[b] **n (%)**	p-value[c]
**Health profession**	***48 (6.3)***	***717 (93.7)***	0.014	***43 (5.7)***	***714 (94.3)***	1.000
Obstetricians/Gynaecologists	9 (3.2)	269 (96.8)		16 (5.7)	263 (94.3)	
Midwives	39 (8.0)	448 (92.0)		27 (5.6)	451 (94.4)	
**Level of health facility**	***48 (6.3)***	***717 (93.7)***	0.097	***43 (5.7)***	***714 (94.3)***	0.047
National hospital	6 (4.5)	126 (95.5)		3 (2.3)	129 (97.7)	
Provincial hospital	15 (9.1)	149 (90.9)		15 (9.2)	148 (90.8)	
District hospital	23 (5.2)	416 (94.8)		22 (5.1)	410 (94.9)	
Maternity home	4 (13.3)	26 (86.7)		3 (10.0)	27 (90.0)	
**Performing ultrasound**[d]	***48 (6.3)***	***716 (93.7)***	0.034	***43 (5.7)***	***713 (94.3)***	0.950
Yes	7 (3.2)	215 (96.8)		12 (5.4)	211 (94.6)	
No	41 (7.6)	501 (92.4)		31 (5.8)	502 (94.2)	
	**(More) midwives trained in ultrasound**[a]					
	Not at all or Not very much[b]	A fair amount or A great deal[b]	p-value[c]			

For peer review only

	n (%)	n (%)	
**Health profession**	***277 (37.5)***	***462 (62.5)***	<0.001
Obstetricians/Gynaecologists	165 (60.7)	107 (39.3)	
Midwives	112 (24.0)	355 (76.0)	
**Level of health facility**	***277 (37.5)***	***462 (62.5)***	0.003
National hospital	50 (38.2)	81 (61.8)	
Provincial hospital	79 (49.4)	81 (50.6)	
District hospital	139 (33.4)	277 (66.6)	
Maternity home	9 (28.1)	23 (71.9)	
**Performing ultrasound**[d]	***277 (37.5)***	***461 (62.5)***	<0.001
Yes	135 (62.2)	82 (37.8)	
No	142 (27.3)	379 (72.7)	

[a]Item in questionnaire: "What do you believe would improve the utilisation of ultrasound at your clinic/work place?"
[b]The response option *don´t know* was excluded from analyses
[c]Pearson's Chi-Square test for categorical variables. Yates' continuity correction was used for 2 by 2 tables.
[d]Performing obstetric ultrasound examinations

For peer review only

Discussion

The main findings of this study are that access to obstetric ultrasound was generally reported as satisfactory regardless of participants' health profession and health facility level. Participants reported an average of almost six ultrasound examinations as medically indicated in an uncomplicated pregnancy in contrast to the three ultrasound examinations that are recommended in the Vietnamese national guidelines. Obstetricians/gynaecologists reported high self-rated skill levels for most obstetric ultrasound examinations, although one third of all health professionals reported that "lack of ultrasound training sometimes leads to suboptimal pregnancy management". "Better quality of ultrasound machines", "more training for health professionals' currently performing ultrasound" and "more physicians trained in ultrasound" were reported as factors that could improve the utilisation of ultrasound to the greatest extent. For participants at health facilities in rural and semi-urban areas compared to those in urban areas, the great majority reported that "maternity care in my country would improve if midwives were qualified to perform basic ultrasound examinations".

The role of obstetric ultrasound

Obstetric ultrasound is one of the most important technological advances in pregnancy surveillance [28]. The majority of health professionals in our study agreed or strongly agreed with the statement "ultrasound is decisive in pregnancy management" but unexpectedly one third of our participants were neutral or disagreed with this statement. Obstetricians/gynaecologists who mainly performed ultrasound reported lower agreement with the above statement than midwives who mainly did not perform ultrasound examinations. CROCUS-study findings in Rwanda also demonstrated that health professionals not themselves performing ultrasounds have more liberal attitudes towards ultrasound use then health professionals performing these examinations [29]. Health

For peer review only

professionals sometimes fear that "routinisation" of ultrasound as an unquestioned and integral part of pregnancy management can exert a negative impact on pregnant women's informed consent, and that its increasing use for fetal examination may have consequences for how disability is viewed in society [25 26 30]. The use of ultrasound may also reduce attention on important clinical parameters such as measurement of blood pressure and proteinuria [24 31]. Previous research from Vietnam has shown that many ultrasound examinations are performed just for reassurance of fetal wellbeing [12]. Although problems of access to obstetric ultrasound exist in many low-resource countries, inappropriate use of ultrasound examinations with no effect on pregnancy outcomes still occurs. In a study from Uganda, more than half of all ultrasound examinations performed were classified as inappropriate, for example dating of pregnancy in a suboptimal gestational week or requesting an ultrasound without medical indication [32]. The Ministry of Health in Vietnam recommends three ultrasound examinations including one examination also in the third trimester. However, a routine late pregnancy ultrasound in unselected or low-risk populations has been reported to have no benefit for mother or baby [33]. It is known that Vietnamese women generally undergo many ultrasound examinations during pregnancy [12]. Although it is well known that ultrasound examinations hold a strong appeal for pregnant women [34], the high number of examinations that pregnant women receive seems also to be supported by the ultrasound operators, as the participants in this study considered that twice as many ultrasound examinations were medically indicated compared to the recommendations by the Ministry of Health, Vietnam [8]. Further, participants' report of a mean number of almost six ultrasound examinations to be medically indicated is also in great contrast to the Cochrane review supporting the 2016 WHO ANC guideline recommending a single ultrasound examination before 24 weeks of gestation [2 35]. Health professionals performing ultrasound in our study also reported performing up to 100 scans per day per ultrasound operator. High workload can lead to inadequate provision of information

For peer review only

to women by the physician, including about indication for ultrasound and the results of the ultrasound examination [12 36]. In a broader perspective, non-medical ultrasound examinations during pregnancy consume resources unnecessarily, with negative impact on other maternal health care services. Issuing medical guidelines stating clear indications for ultrasound surveillance during pregnancy are therefore important and may contribute to more appropriate allocation of resources within the health care system in Vietnam.

Access to obstetric ultrasound

Health professionals in our study generally reported satisfactory access to obstetric ultrasound when needed in their own workplace. Access to ultrasound has increased significantly in many resource-limited settings although there are still large differences in access within and between countries [37]. Our study was performed in the area around Hanoi, i.e. the capital of Vietnam, and it is plausible to believe that access to obstetric ultrasound is higher in this region than in other parts of Vietnam. In Hanoi, ultrasound scans are easily accessible and affordable for most pregnant women both within the public and private health care systems. Increased ANC attendance and health facility delivery rates have been seen in Vietnam in recent years, but also increased inequities in maternity care utilisation [38 39], primarily among women with multiple socioeconomic vulnerabilities [38].

Ultrasound operators' skills

Participants performing ultrasound in our study reported high or intermediate skill levels for the majority of the specified ultrasound examinations. Proper training of health professionals performing ultrasound is critically important [28] especially since ultrasound is operator dependent to a large extent [4]. Further, ultrasound training should include ethics, discuss use and misuse of the ultrasound tool in addition to the quality of ultrasound performance and

For peer review only

clinical implications of its use [28], to ensure maximum diagnostic utility and high levels of sensitivity and specificity [4].

In our study, one third of all health professionals believed that insufficient training of the ultrasound operator sometimes leads to suboptimal pregnancy management. Another study from Vietnam reports that physicians experience less knowledge about fetal anomalies, lack advanced training and do not have appropriate equipment and professional protocols that could support their practice in performing obstetric ultrasound [40]. Inadequately skilled health professionals may cause inadvertent harm, for example, by providing false-positive diagnosis where termination of pregnancy may be an option. Alternatively, false-negative information because of inadequate ability to recognise the signs of important diagnoses can result in parents not being offered further investigations and being inappropriately reassured that everything is normal [41].

Improving utilisation of ultrasound

Our results indicate an increased number of physicians to become appropriately educated in ultrasound examinations, regular in-service training sessions and better quality of ultrasound equipment. Lack of training of health care providers has been seen as the most common barrier to regular ultrasound use, although lack of equipment and maintenance and costs for machines also are explanatory factors [42]. A review of ultrasound training in low and middle income countries (LMICs) shows that health professionals often do not meet the WHO criteria in relation to number of scans, supervision and length of training [43]. The obstacles to ultrasound training in LMICs include lack of time for training because of limited possibilities for absence from the workplace, and the logistics to access qualified teachers [43]. Our results also indicate that health professionals in Vietnam seem to have a very substantial workload. It

For peer review only - http://bmjopen.bmj.com/site/about/guidelines.xhtml

has been shown that training of midwives in basic obstetric ultrasound may significantly improve pregnancy management [44-46]. In our study, the majority of midwives were positive to the idea of training midwives in ultrasound to improve utilisation of ultrasound. For rural and semi-urban hospitals, a majority of all participants were also positive to ultrasound-trained midwives. However, obstetricians/gynaecologists did not support the idea of training midwives in ultrasound as much as participating midwives. As in much of Vietnamese society, health professions are organised hierarchically. Nurses' roles can be seen as primarily carrying out doctors' orders, not to take their own initiatives [47 48], and it may be a plausible explanation for why physicians defend performing ultrasound as their duty. Shortages of nurses and midwives and lack of quality of training [49 50], may also be additional explanations for physicians not supporting the idea of training midwives in obstetric ultrasound.

Strengths and limitations

The strengths of this study include participants recruited from different levels of the health care system in urban, semi-urban and rural areas of Hanoi. We believe that the wide range of health facilities is likely to be representative for the Vietnamese health care system. The research team comprised two Vietnamese researchers familiar with the setting and the health care system which strengthens the interpretation of data. An additional strength was that four experienced Vietnamese data collectors, familiar with the setting, collected all data. One limitation of this study may be the translation of the questionnaire from English to Vietnamese, with the risk of losing the intended meaning of questions and statements. However, measures to reduce this risk were implemented, including back-translation of the questionnaire. Another limitation might be the unequal distribution between the categories of physicians and midwives in this sample. Although we aimed for equal numbers of each category of health professional, as assessed in the power calculation, all eligible physicians

For peer review only

and midwives working the day of data collection were included in the study. This resulted in a higher number of midwives. Since there is a lack of previous studies within this research domain, the power calculation was based on assumptions of proportions for one outcome variable in relation to one background variable. The assumptions in the power calculations therefore mean uncertainty of the required study sample. However, the results indicate that the sample size was probably adequate for investigating our questions of interest. There was no question in the questionnaire about whether the participant had received any formal ultrasound training and this may be considered a limitation. In Vietnam, it is widely recognised that participants in studies receive payment. All participants were therefore paid with a small sum for answering the questionnaire which may theoretically have affected the willingness of participation in the study.

Conclusions

Obstetric ultrasound is used as an integral part of antenatal care at all selected health facility levels in the region of Hanoi and access to obstetric ultrasound was also reported to be good at all levels. Overall, participants performing obstetric ultrasound reported satisfactory self-rated skill levels. However, report of insufficient ultrasound training resulting in suboptimal pregnancy management in combination with the suggestion for more training to improve utilisation of ultrasound, indicates a need for additional in-service training of ultrasound operators. The proposal of educating midwives in obstetric ultrasound needs to be further evaluated.

Acknowledgements

Our sincere thanks to the participating health professionals for sharing their time and experiences and for the support by the heads of the Departments of Obstetrics and

For peer review only - http://bmjopen.bmj.com/site/about/guidelines.xhtml

Gynaecology at the selected health facilities. Thanks go to the data collectors Do Nam Khanh, Nguyen Huyen Tram, Le Vu Thuy Huong and Nguyen Thi Hue for excellent performance of the data collection. We also thank Annika Åhman, Uppsala University, Sweden, for valuable contributions to the questionnaire and Gabriel Granåsen, Umeå University, Sweden for statistical support. We acknowledge the support received from Hanoi Medical University, Hanoi, Vietnam and Umeå University, Umeå, Sweden.

Abbreviations

CROCUS: Cross Country Ultrasound study; ANC: Antenatal care; WHO: World Health Organization; VHW: Village health worker; CHS: Community health centre level; SD: Standard deviation; OR: Odds ratio; CI: Confidence intervals; LMICs: Low and middle income countries.

Footnotes

Authors' contributions

IM is principal investigator of the CROCUS study. SH, LPT, KE, JN, RS, HLK, MN and IM designed the study. SH, KE and IM were the main responsible for developing the questionnaire with input from all co-authors. LPT, PHD, SH, KE and IM organised and performed the data collection together with data collectors. SH conducted the analyses in collaboration with IM, LPT and PHD. SH drafted the primary manuscript in collaboration with IM. All authors have contributed to and approved the final manuscript, tables and figures.

Funding

For peer review only

This research was made possible by grants from Umeå University, Västerbotten County Council, The Swedish Research Council, Sweden (2014-2672), the Swedish Research Council for Health, Working Life and Welfare (Forte) and the European Commission under a COFAS Marie Curie Fellowship (2013-2699).

Competing interests

The authors declare that they have no competing interests.

Ethics approval and consent to participate

Ethical approval was obtained from the Hanoi Medical University Review Board in Bio-Medical Research, reference 141/HMU IRB. The participants received both written and verbal information about the study including that participation was voluntary. Questionnaires were only returned if the participants choose to do so and the return of the questionnaire was taken as evidence of informed consent.

Data sharing statement

The datasets used and/or analysed during the current study are available from the corresponding author on reasonable request.

For peer review only

References

1. Ministry of Health Viet Nam, Partnership for Maternal, Newborn & Child Health, WHO, World Bank and Alliance for Health Policy and Systems Research. Sucess factors for women´s and children´s health: Viet Nam. Geneva: World Health Organisation, 2014.
2. World Health Organization. WHO recommendations on antenatal care for a positive pregnancy experience. Geneva, 2016.
3. Salomon LJ, Alfirevic Z, Berghella V, et al. on behalf of the ISUOG Clinical Standards Committe. Practice guidelines for performence of the routine mid-trimester fetal ultrasound scan. *Ultrasound Obstet Gynecol (2010)* doi: 10.1002/uog.8831
4. Stanton K, Mwanri L. Global Maternal and Child Health Outcomes: The Role of Obstetric Ultrasound in Low Resource Settings. *World Journal of Preventive Medicine* 2013;1(3):22-29.
5. Duc NHC, Nakamura K, Kizuki M, et al. Trends in inequalities in utilization of reproductive health services from 2000 to 2011 in Vietnam. *Journal of Rural Medicine : JRM* 2015;10(2):65-78. doi: 10.2185/jrm.2902
6. Goland E, Hoa DT, Malqvist M. Inequity in maternal health care utilization in Vietnam. *International journal for equity in health* 2012;11:24. doi: 10.1186/1475-9276-11-24
7. Edvardsson K, Graner S, Thi LP, et al. 'Women think pregnancy management means obstetric ultrasound': Vietnamese obstetricians' views on the use of ultrasound during pregnancy. *Glob Health Action* 2015;8(1):28405. doi: 10.3402/gha.v8.28405
8. Ministry of Health Viet Nam. National guidelines for reproductive healthcare, 2016.
9. World Health Organization. WHO antenatal care randomized trial: manual for the implementation of the new model. Geneva: World Health Organization, 2002.
10. UNICEF and General Statistics Office. Viet Nam Multiple Indiciator Cluster Survey 2014, Final Report. Ha Noi, Viet Nam, 2015.
11. UNFPA. Improvning the Quality of Reproductive Health Care Services in Viet Nam. The Role of National Standards and Guidelines for Reproductive Health Care Services. Ha Noi, 2007.
12. Gammeltoft T, Nguyen HT. The commodification of obstetric ultrasound scanning in Hanoi, Viet Nam. *Reprod Health Matters* 2007;15(29):163-71. doi: 10.1016/S0968-8080(06)29280-2 [published Online First: 2007/05/22]
13. Vujicic M, Shengelia B, Alfano M, et al. Physician shortages in rural Vietnam: using a labor market approach to inform policy. *Soc Sci Med* 2011;73(7):970-7. doi: 10.1016/j.socscimed.2011.06.010
14. Tran TK, Nguyen CTK, Nguyen HD, et al. Urban - rural disparities in antenatal care utilization: a study of two cohorts of pregnant women in Vietnam. *BMC health services research* 2011;11:120-20. doi: 10.1186/1472-6963-11-120
15. The World Bank, Vietnam. http://data.worldbank.org, 2017.
16. WHO and the Ministry of Health Viet Nam. Health Service Delivery Profile Viet Nam, 2012.
17. Ministry of Health, Viet Nam. Department of Planning and Finance and Health Insurance. Social health insurance scheme in Vietnam. Achievements & Challanges. Tokyo, 2016.
18. Raven J, Liu X, Hu D, et al. Improving neonatal health in remote rural areas in China and Vietnam: synthesis report. Liverpool School of Tropical Medicine, Liverpool UK, 2015
19. Ahman A, Kidanto HL, Ngarina M, et al. 'Essential but not always available when needed' - an interview study of physicians' experiences and views regarding use of obstetric ultrasound in Tanzania. *Glob Health Action* 2016;9(1):31062. doi: 10.3402/gha.v9.31062

For peer review only

20. Edvardsson K, Ntaganira J, Ahman A, et al. Physicians' experiences and views on the role of obstetric ultrasound in rural and urban Rwanda: a qualitative study. *Trop Med Int Health* 2016;21(7):895-906. doi: 10.1111/tmi.12718

21. Holmlund S, Ntaganira J, Edvardsson K, et al. Improved maternity care if midwives learn to perform ultrasound: a qualitative study of Rwandan midwives' experiences and views of obstetric ultrasound. *Glob Health Action* 2017;10(1):1350451. doi: 10.1080/16549716.2017.1350451

22. Åhman A, Edvardsson K, Lesio Kidanto H, et al. 'Without ultrasound you can't reach the best decision' – Midwives' experiences and views of the role of ultrasound in maternity care in Dar Es Salaam, Tanzania. *Sexual & Reproductive Healthcare* 2018;15:28-34. doi: https://doi.org/10.1016/j.srhc.2017.11.007

23. Edvardsson K, Small R, Persson M, et al. 'Ultrasound is an invaluable third eye, but it can't see everything': a qualitative study with obstetricians in Australia. *BMC Pregnancy Childbirth* 2014;14:363. doi: 10.1186/1471-2393-14-363

24. Ahman A, Persson M, Edvardsson K, et al. Two sides of the same coin--an interview study of Swedish obstetricians' experiences using ultrasound in pregnancy management. *BMC Pregnancy Childbirth* 2015;15:304. doi: 10.1186/s12884-015-0743-5

25. Edvardsson K, Mogren I, Lalos A, et al. A routine tool with far-reaching influence: Australian midwives' views on the use of ultrasound during pregnancy. *BMC Pregnancy Childbirth* 2015;15:195. doi: 10.1186/s12884-015-0632-y

26. Edvardsson K, Lalos A, Ahman A, et al. Increasing possibilities - Increasing dilemmas: A qualitative study of Swedish midwives' experiences of ultrasound use in pregnancy. *Midwifery* 2016;42:46-53. doi: 10.1016/j.midw.2016.09.009

27. Edvardsson K, Small R, Lalos A, et al. Ultrasound's 'window on the womb' brings ethical challenges for balancing maternal and fetal health interests: obstetricians' experiences in Australia. *BMC Med Ethics* 2015;16:31. doi: 10.1186/s12910-015-0023-y

28. Kongnyuy EJ, van den Broek N. The use of ultrasonography in obstetrics in developing countries. *Trop Doct* 2007;37(2):70-2. doi: 10.1177/004947550703700203

29. Holmlund S, Ntaganira J, Edvardsson K, et al. Health professionals' experiences and views on obstetric ultrasound in Rwanda: A cross-sectional study. *PLOS ONE* 2018;13(12):e0208387. doi: 10.1371/journal.pone.0208387

30. Edvardsson K, Åhman A, Fagerli TA, et al. Norwegian obstetricians' experiences of the use of ultrasound in pregnancy management. A qualitative study. *Sexual & Reproductive Healthcare* 2018;15:69-76. doi: https://doi.org/10.1016/j.srhc.2017.12.001

31. Edvardsson K, Graner S, Thi LP, et al. 'Women think pregnancy management means obstetric ultrasound': Vietnamese obstetricians' views on the use of ultrasound during pregnancy. *Glob Health Action* 2015;8:28405. doi: 10.3402/gha.v8.28405

32. Gonzaga MA, Kiguli-Malwadde E, Businge F, et al. Utilisation of obstetric sonography at a peri-urban health centre in Uganda. *The Pan African Medical Journal* 2010;7:24.

33. Bricker L, Medley N, Pratt JJ. Routine ultrasound in late pregnancy (after 24 weeks' gestation). *Cochrane Database of Systematic Reviews* 2015(6) doi: 10.1002/14651858.CD001451.pub4

34. Garcia J, Bricker L, Henderson J, et al. Women's views of pregnancy ultrasound: a systematic review. *Birth* 2002;29(4):225-50. [published Online First: 2002/11/15]

35. Whitworth M, Bricker L, Mullan C. Ultrasound for fetal assessment in early pregnancy. *Cochrane Database Syst Rev* 2015(7):CD007058. doi: 10.1002/14651858.CD007058.pub3

36. Mensah YB, Nkyekyer K, Mensah K. The Ghanaian Woman's Experience and Perception of Ultrasound Use in Antenatal Care. *Ghana Medical Journal* 2014;48(1):31-38.

For peer review only - http://bmjopen.bmj.com/site/about/guidelines.xhtml

37. Sippel S, Muruganandan K, Levine A, et al. Review article: Use of ultrasound in the developing world. *International Journal of Emergency Medicine* 2011;4:72-72. doi: 10.1186/1865-1380-4-72
38. Van Minh H, Oh J, Giang KB, et al. Multiple vulnerabilities and maternal healthcare in Vietnam: findings from the Multiple Indicator Cluster Surveys, 2000, 2006, and 2011. *Global Health Action* 2016;9:10.3402/gha.v9.29386. doi: 10.3402/gha.v9.29386
39. Malqvist M, Lincetto O, Du NH, et al. Maternal health care utilization in Viet Nam: increasing ethnic inequity. *Bull World Health Organ* 2013;91(4):254-61. doi: 10.2471/BLT.12.112425
40. Gammeltoft T, Nguyen HT. Fetal conditions and fatal decisions: ethical dilemmas in ultrasound screening in Vietnam. *Soc Sci Med* 2007;64(11):2248-59. doi: 10.1016/j.socscimed.2007.02.015
41. Howe D. Ethics of prenatal ultrasound. *Best Practice & Research Clinical Obstetrics & Gynaecology* 2014;28(3):443-51. doi: https://doi.org/10.1016/j.bpobgyn.2013.10.005
42. Shah S, Bellows BA, Adedipe AA, et al. Perceived barriers in the use of ultrasound in developing countries. *Critical Ultrasound Journal* 2015;7(1):11. doi: 10.1186/s13089-015-0028-2
43. LaGrone LN, Sadasivam V, Kushner AL, et al. A review of training opportunities for ultrasonography in low and middle income countries. *Trop Med Int Health* 2012;17(7):808-19. doi: 10.1111/j.1365-3156.2012.03014.x
44. Swanson JO, Kawooya MG, Swanson DL, et al. The diagnostic impact of limited, screening obstetric ultrasound when performed by midwives in rural Uganda. *J Perinatol* 2014;34(7):508-12. doi: 10.1038/jp.2014.54 [published Online First: 2014/04/05]
45. Nathan RO, Swanson JO, Swanson DL, et al. Evaluation of Focused Obstetric Ultrasound Examinations by Health Care Personnel in the Democratic Republic of Congo, Guatemala, Kenya, Pakistan, and Zambia. *Current Problems in Diagnostic Radiology* 2017;46(3):210-15. doi: https://doi.org/10.1067/j.cpradiol.2016.11.001
46. Bentley S, Hexom B, Nelson BP. Evaluation of an Obstetric Ultrasound Curriculum for Midwives in Liberia. *J Ultrasound Med* 2015;34(9):1563-8. doi: 10.7863/ultra.15.14.08017
47. Nancy Waxler-Morrison JMA, Elisabeth Richardson, Natalie A. Chambers. Cross-Cultural Caring: A handbook for Health Professionals. 2nd ed. Vancouver, Canada: UBC Press 2011.
48. Jones PS, O'Toole MT, Hoa N, et al. Empowerment of nursing as a socially significant profession in Vietnam. *Journal of Nursing Scholarship* 2000;32(3):317-21. doi: 10.1111/j.1547-5069.2000.00317.x
49. The World Bank. World Health Organization's Global Health Workforce Statistics, OECD, supplemented by country data 2013 [cited 2017 2017-01.15]. Available from: www.data.worldbank.org.
50. World Health Organization. Regional Office for the Western Pacific. Human resources for health: action framework for the Western Pacific Region (2011-2015). Manila, 2012.

For peer review only

Figure legend

Fig. 1. Obstetricians/Gynaecologists' self-rated skills for specified ultrasound examinations (n=227). Reported skill levels are presented with proportions

For peer review only

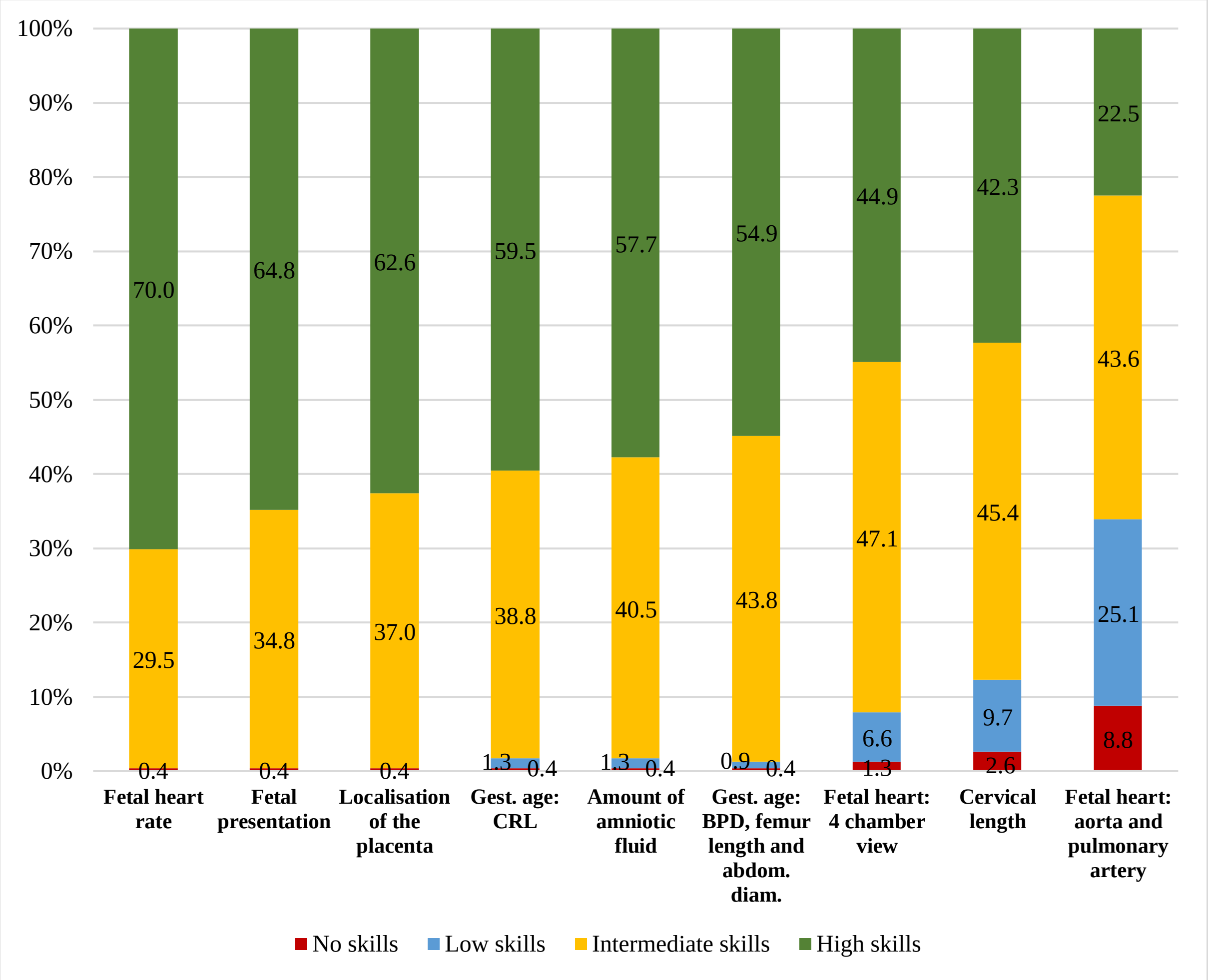
100%
90%
80%
70%
60%
50%
40%
30%
20%
10%
0%
70.0
29.5
0.4
64.8
34.8
0.4
62.6
37.0
0.4
59.5
38.8
1.3
0.4
57.7
40.5
1.3
0.4
54.9
43.8
0.9
0.4
44.9
47.1
6.6
1.3
42.3
45.4
9.7
2.6
22.5
43.6
25.1
8.8
Fetal heart rate
Fetal presentation
Localisation of the placenta
Gest. age: CRL
Amount of amniotic fluid
Gest. age: BPD, femur length and abdom. diam.
Fetal heart: 4 chamber view
Cervical length
Fetal heart: aorta and pulmonary artery
No skills
Low skills
Intermediate skills
High skills

STROBE 2007 (v4) Statement—Checklist of items that should be included in reports of *cross-sectional studies*

Section/Topic	Item #	Recommendation	Reported on page #
**Title and abstract**	1	(*a*) Indicate the study's design with a commonly used term in the title or the abstract	1
		(*b*) Provide in the abstract an informative and balanced summary of what was done and what was found	3-4
**Introduction**			
Background/rationale	2	Explain the scientific background and rationale for the investigation being reported	6-7
Objectives	3	State specific objectives, including any prespecified hypotheses	6-7
**Methods**			
Study design	4	Present key elements of study design early in the paper	9
Setting	5	Describe the setting, locations, and relevant dates, including periods of recruitment, exposure, follow-up, and data collection	8-11
Participants	6	(*a*) Give the eligibility criteria, and the sources and methods of selection of participants	10
Variables	7	Clearly define all outcomes, exposures, predictors, potential confounders, and effect modifiers. Give diagnostic criteria, if applicable	11-13
Data sources/ measurement	8*	For each variable of interest, give sources of data and details of methods of assessment (measurement). Describe comparability of assessment methods if there is more than one group	13
Bias	9	Describe any efforts to address potential sources of bias	9-10
Study size	10	Explain how the study size was arrived at	9
Quantitative variables	11	Explain how quantitative variables were handled in the analyses. If applicable, describe which groupings were chosen and why	11-13
Statistical methods	12	(*a*) Describe all statistical methods, including those used to control for confounding	13
		(*b*) Describe any methods used to examine subgroups and interactions	
		(*c*) Explain how missing data were addressed	
		(*d*) If applicable, describe analytical methods taking account of sampling strategy	
		(*e*) Describe any sensitivity analyses	
**Results**			

For peer review only

Participants	13*	(a) Report numbers of individuals at each stage of study—eg numbers potentially eligible, examined for eligibility, confirmed eligible, included in the study, completing follow-up, and analysed	14-15
		(b) Give reasons for non-participation at each stage	10
		(c) Consider use of a flow diagram	
Descriptive data	14*	(a) Give characteristics of study participants (eg demographic, clinical, social) and information on exposures and potential confounders	14-15
		(b) Indicate number of participants with missing data for each variable of interest	12, 14-19
Outcome data	15*	Report numbers of outcome events or summary measures	14-21
Main results	16	(*a*) Give unadjusted estimates and, if applicable, confounder-adjusted estimates and their precision (eg, 95% confidence interval). Make clear which confounders were adjusted for and why they were included	14-21
		(*b*) Report category boundaries when continuous variables were categorized	
		(*c*) If relevant, consider translating estimates of relative risk into absolute risk for a meaningful time period	
Other analyses	17	Report other analyses done—eg analyses of subgroups and interactions, and sensitivity analyses	
**Discussion**			
Key results	18	Summarise key results with reference to study objectives	22, 27
Limitations	19	Discuss limitations of the study, taking into account sources of potential bias or imprecision. Discuss both direction and magnitude of any potential bias	26-27
Interpretation	20	Give a cautious overall interpretation of results considering objectives, limitations, multiplicity of analyses, results from similar studies, and other relevant evidence	22-26
Generalisability	21	Discuss the generalisability (external validity) of the study results	26-27
**Other information**			
Funding	22	Give the source of funding and the role of the funders for the present study and, if applicable, for the original study on which the present article is based	29

*Give information separately for cases and controls in case-control studies and, if applicable, for exposed and unexposed groups in cohort and cross-sectional studies.

Note: An Explanation and Elaboration article discusses each checklist item and gives methodological background and published examples of transparent reporting. The STROBE checklist is best used in conjunction with this article (freely available on the Web sites of PLoS Medicine at http://www.plosmedicine.org/, Annals of Internal Medicine at http://www.annals.org/, and Epidemiology at http://www.epidem.com/). Information on the STROBE Initiative is available at www.strobe-statement.org.

For peer review only
